# ﻿Additions to the taxonomy of the *Auriculariales* (Basidiomycota) with pedunculate basidia

**DOI:** 10.3897/mycokeys.120.155492

**Published:** 2025-08-19

**Authors:** Viacheslav Spirin, Vera Malysheva, Ilya Viner, Renato Lúcio Mendes Alvarenga, Tine Grebenc, Gérald Gruhn, Anton Savchenko, Django Grootmyers, Leif Ryvarden, Josef Vlasák, Karl-Henrik Larsson, R. Henrik Nilsson

**Affiliations:** 1 Department of Biological and Environmental Sciences, Box 463, University of Gothenburg, 405 30 Gothenburg, Sweden; 2 Finnish Museum of Natural History, University of Helsinki, PO Box 7, 00014 Helsinki, Finland; 3 Shatelena str. 20/75, 194021 St. Petersburg, Russia; 4 Departamento de Micologia, Universidade Federal de Pernambuco (UFPE), Avenida da Engenharia s/n, Recife, Pernambuco, 50740-600, Brazil; 5 Slovenian Forestry Institute, Večna pot 2, 1000 Ljubljana, Slovenia; 6 Office National des Forêts, 5 avenue Mirandol, 48000 Mende, France; 7 Naturalis Biodiversity Center, Darwinweg 2, 2333 CR Leiden, Netherlands; 8 Department of Ecology and Evolutionary Biology, University of Tennessee, 1406 Circle Drive, Knoxville, Tennessee 37996, USA; 9 Institute of Biological Sciences, University of Oslo, P.O. Box 1045, Blindern, N-0316 Oslo, Norway; 10 Biology Centre, Academy of Sciences of the Czech Republic, Branišovská 31, CZ 37005, České Budějovice, Czech Republic; 11 Natural History Museum, University of Oslo, P.O. Box 1172, Blindern, 0318 Oslo, Norway; 12 Gothenburg Global Biodiversity Centre, University of Gothenburg, Box 461, 40530 Gothenburg, Sweden

**Keywords:** heterobasidiomycetes, phylogeny, soil sequences, taxonomy

## Abstract

In the present paper, we revise the taxonomy of the Auriculariales having pedunculate (stalked) basidia. In total, sixteen new species from Europe, East Asia, North and South America, and tropical Africa are described. They are classified among the genera *Hydrophana*, *Mycostilla*, *Myxarium*, *Protoacia*, *Protohydnum*, and *Protomerulius*. In addition, the generic affiliation of eleven extant species is re-established based on phylogenetic and/or morphological evidence. A new genus, *Elmericium*, is introduced to accommodate a crust-like fungus, *E.alabastrinum*, from East Asia; phylogenetic data place it in the vicinity of the anatomically similar poroid genera *Elmerina* and *Protodaedalea* (Auriculariaceae). The generic description of *Protohydnum* is amended; in its current scope, the genus encompasses several species formerly assigned to *Bourdotia*, *Ductifera*, and *Exidiopsis*. Available environmental data point to a wider distribution of some *Protomerulius* spp. and greater species diversity in the genus than currently surmised from physical fungal samples.

## ﻿Introduction

The Auriculariales Bromhead (Agaricomycetes Doweld, Basidiomycota Whittaker ex R.T. Moore) are an order of wood-inhabiting heterobasidiomycetes with diversely structured basidia. The great majority of species have four-, more rarely two-celled, longitudinally septate basidia (e.g., *Exidia* Fr. and *Pseudohydnum* P. Karst.); at the same time, the order also encompasses fungi with transversally (*Auricularia* Bull.) or obliquely septate (*Patouillardina* Bres.) basidia, or possessing one-celled, non-septate basidia (*Oliveonia* Donk) (Roberts 1999; Weiß and Oberwinkler 2001; Weiß et al. 2004). Among the species with longitudinally septate basidia, a large group of taxa have the so-called sphaeropedunculate or myxarioid basidia – in this case, two or four basidial cells are located at the top of a pronounced, stipe-like cell (the “enucleate stalk”). Wells (1964) first argued that sphaeropedunculate basidia represent a taxonomically important morphological trait. However, subsequent research provided different views on the taxonomic value of these structures (Bandoni 1984; Wells 1994; Roberts 1998).

Recent DNA-based studies indicated that the presence of pedunculate (stalked) basidia is informative for the generic reclassification of the Auriculariales, although its value is not absolute (Wells et al. 2004; Sotome et al. 2014; Malysheva and Spirin 2017; Malysheva et al. 2018; Spirin et al. 2018a, 2019a, b, c). In the present paper, we expand the existing knowledge of the Auriculariales with stalked basidia: both taxa with sphaeropedunculate (myxarioid) basidia (e.g., *Myxarium*, *Protomerulius*) and those with petiolate basidia (e.g., *Bourdotia*, *Protohydnum*) are dealt with below. To supplement our data on the ecology and distribution of the species, we utilise available (as a rule, yet unnamed) environmental and soil sequences from public repositories.

## ﻿Material and methods

### ﻿Morphological study

Specimens from herbaria H, GB, S, O, LE, FH, PC, K, BPI, NY, HBG, LIP, LY, TAAM, CWU, LJF, TUF, and URM were studied; herbarium acronyms are given according to Index Herbariorum (https://sweetgum.nybg.org/science/ih). Microscopic routine and measuring techniques follow Miettinen et al. (2006), and terminology follows Malysheva et al. (2018). All measurements were made in Cotton Blue. No reactions with Melzer’s reagent (amyloid or dextrinoid structures) are known in the studied group. Basidiospores of all species dealt with below are hyaline, smooth, and thin-walled; these features are not mentioned in the species descriptions.

### ﻿DNA extraction and amplification

For DNA extraction protocols, PCR, and sequencing of target loci performed at the Finnish Museum of Natural History, University of Helsinki (Finland), see Viner et al. (2021a) and references therein; for sequencing protocols performed at the Komarov Botanical Institute RAS (Saint Petersburg, Russia), see Spirin et al. (2024a). In this study, we amplified the complete nuclear ribosomal DNA ITS1–5.8S–ITS2 (ITS) and partial LSU regions using standard primers (Table 1). Chromatograms were edited and assembled in MEGA X (Kumar et al. 2018); chromatograms exhibiting length variation due to indels were interpreted and assembled as described in Viner et al. (2021b). Additional sequences used in our analyses were retrieved from partial genomes following the methods outlined in Spirin et al. (2022). All newly generated sequences of sufficient quality, including those not used in the phylogenetic analyses, have been submitted to GenBank (Table 2).

**Table 1.** Primers used in this study.

Primer name	Sequence	Target DNA locus	Binding site	Direction	Reference
ITS1F	CTTGGTCATTTAGAGGAAGTAA	ITS, ITS1	18S	fwd	Gardes and Bruns 1993
ITS5	GGAAGTAAAAGTCGTAACAAGG	ITS, ITS1	18S	fwd	White et al. 1990
ITS2	GCTGCGTTCTTCATCGATGC	ITS1	5.8S	rev	White et al. 1990
ITS3	GCATCGATGAAGAACGCAGC	ITS2	5.8S	fwd	White et al. 1990
ITS4	TCCTCCGCTTATTGATATGC	ITS, ITS2	28S	rev	White et al. 1990
LR22	CCTCACGGTACTTGTTCGCT	ITS	28S	rev	Vilgalys lab, Duke University (https://sites.duke.edu/vilgalyslab/ files/2017/08/rDNA-primers-for-fungi.pdf)
JS1	CGCTGAACTTAAGCATAT	28S	28S	fwd	Landvik 1996
LR7	TACTACCACCAAGATCT	28S	28S	rev	Hopple and Vilgalys 1994
LR5	TCCTGAGGGAAACTTCG	28S	28S	rev	Hopple and Vilgalys 1994

**Table 2.** DNA sequences obtained for the present study.

Species	Specimen / herbarium	Country of origin (ISO 3166 code)	GenBank / UNITE accession number	
			ITS	LSU
*Elmericiumalabastrinum*	Spirin 5311 (H)	RU-KHA	PV241605	PV241619
*Endoperplexadartmorica*	Spirin 11781 (O)	NO	MT235621	MT235602
*E.dartmorica*	Spirin 11783	NO	MT235620	
*Exidiopsisgloeophora*	J. Nordén 9609 (O)	NO	PV241611	PV241624
*Exidiopsissuccinea*	Spirin 5836 (H)	RU-LEN	PV241617	PV241632
*E.succinea*	Spirin 7958 (H)	RU-KHA	PV241618	
*Hydrophanafessula*	Viner 2021/289 (H)	FI	PV394849	PV394849
*H.trichiesiana*	Gruhn 18-573 (LIP)	GF	PV462016	PV460969
*Mycostillachromatica*	Spirin 14839 (H)	SI	PV394846	PV394846
*M.vermiformis*	Spirin 15335 (H)	FR	PV394858	
*Myxariumcinnamomescens*	Bulakh LE F-347687	RU-SAK	PV241609	
*M.crystallinum*	Viner 2023/26 (H)	FI	PV394865	PV394865
*M.denticulatum*	Spirin 17365 (GB)	SE	PV394879	PV394879
*M.evanidum*	Spirin 15193 (H)	FI	PV394878	
*M.evanidum*	Spirin 17761 (GB)	SE	PV394881	
*M.fugacissimum*	Spirin 17971 (H)	FR	PV394883	
*M.grilletii*	Spirin 16479 (H)	FR	PV394875	
*M.guianense*	Gruhn 18-559 (LIP)	GF	PV394847	PV394847
*M.hyalinum*	Spirin 17044 (GB)	SE	PV394867	
*M.legonii*	Spirin 16419 (H)	FR	PV394877	
*M.minutissimum*	Spirin 16683 (H)	SI	PV394873	
*M.minutissimum*	Spirin 17270 (GB)	SE	PV394874	
*M.podlachicum*	Spirin 16589 (GB)	SE	PV394866	
*M.varium*	Spirin 15646 (H)	FR	PV394876	
*Oliveoniafibrillosa*	Spirin 8257 (H)	US-WA	MT235628	MT235607
*Protoaciacrispans*	Spirin 17688 (GB)	SE	PV394880	
*P.reliqua*	Grootmyers 21072014 (H)	US-TN	PV394850	PV394850
*Protohydnumalbum*	Burdsall 9422 (LE 23063)	US-OH	PV241608	PV241622
*P.album*	Cain LE 37320	US-IL	PV241607	PV241621
*P.album*	Miettinen 19583 (H)	US-TN	PV241606	PV241620
*P.elevatum*	Bulakh LE F-347688	RU-SAK	PV394861	PV394861
*P.erumpens*	Savchenko 171123/1515 (H)	KE	UDB	UDB
*P.galzinii*	Viner 2022/1023 (H)	ES	PV394871	
*P.galzinii*	Akulov CWU 4564	UA	PV241610	PV241623
*P.lactescens*	Wells TAAM192048	US-CA	PV241612	PV241625
*P.livescens*	Viner 2022/7 (H)	GR	PV394848	PV394848
*P.livescens*	Miettinen 20660 (H)	RO	PV394845	PV394845
*P.livescens*	Spirin 13913 (H)	SI		PV241628
*P.livescens*	Miettinen 15872.2 (H)	ES	PV241614	PV241627
*P.livescens*	Miettinen 15891.1 (H)	ES	PV241615	PV241629
*P.livescens*	Miettinen 16000 (H)	ES	PV241613	PV241626
*P.nudum*	Ryvarden 9435 (O)	KE	PV394856	
*P.ocellatum*	Larsson 15431 (URM)	BR-RO		PV241630
*P.pallidum*	Ryvarden 26180 (O)	ZW	PV241616	PV241631
*Protohydnum* sp.	Vlasák 1808/145 (H)	GF	PV369164	PV383274
*Protomeruliusamiliavi*	Spirin 16165 (H)	FR	PV394854	PV394854
*P.brachysporus*	Spirin 16288 (H)	FR	PV394855	PV394855
*P.commotus*	Spirin 17127 (H)	FR	PV394870	
*P.commotus*	Spirin 13835 (H)	IT	PV394857	
*P.commotus*	Spirin 13617 (H)	CH	PV394851	
*P.deceptorius*	Spirin 14811 (H)	SI	PV394853	PV394853
*P.deceptorius*	Spirin 14592 (H)	SI	PV394852	PV394852
*P.deceptorius*	Spirin 14651 (H)	SI	PV394859	
*P.deceptorius*	Spirin 16764 (H)	SI	PV394863	
*P.deceptorius*	Spirin 16863 (H)	SI	PV394864	
*P.dubius*	Viner 2019/154 (H)	FI	PV394872	
*P.madidus*	Spirin 17851 (H)	FR	PV394882	
*P.madidus*	Spirin 15021 (H)	RO	PV394860	
*P.madidus*	Spirin 16951 (GB)	SE	PV394862	
*P.pertusus*	Kotiranta 22589 (H)	RU-AD	PV394869	
*P.pertusus*	Spirin 12743 (H)	RU-NIZ	PV394868	

### ﻿Phylogenetic analyses

**Figure 1.**
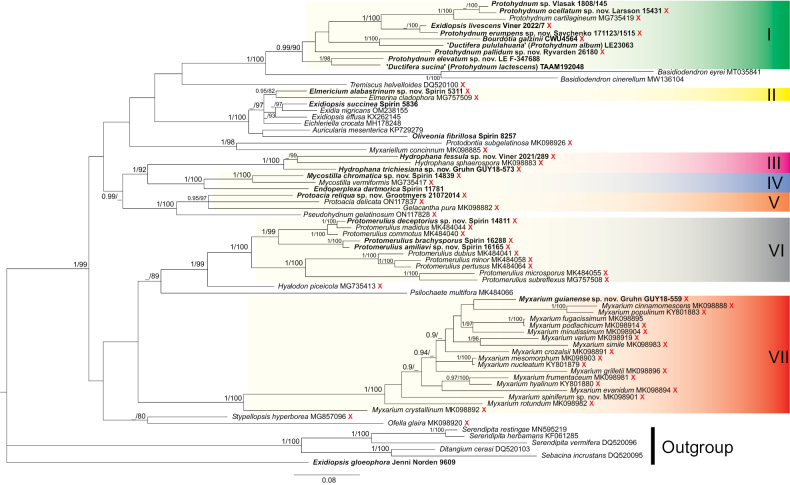
Bayesian majority-rule consensus ITS + LSU-based tree of the Auriculariales overlaid with bootstrap support values from the maximum likelihood analysis. Support values are given as Bayesian posterior probabilities / ML bootstrap percentages. Species with stalked basidia are marked with a red X. I – Protohydnum clade; II – Elmerina clade; III – Hydrophana clade; IV – Endoperplexa – Mycostilla clade; V – Protoacia – Gelacantha clade; VI – Protomerulius clade; VII – Myxarium clade. The Sebacinales (*Ditangium* P. Karst., *Sebacina*, and *Serendipita* P. Roberts spp.) and *Exidiopsisgloeophora* were used as outgroups.

**Figure 2.**
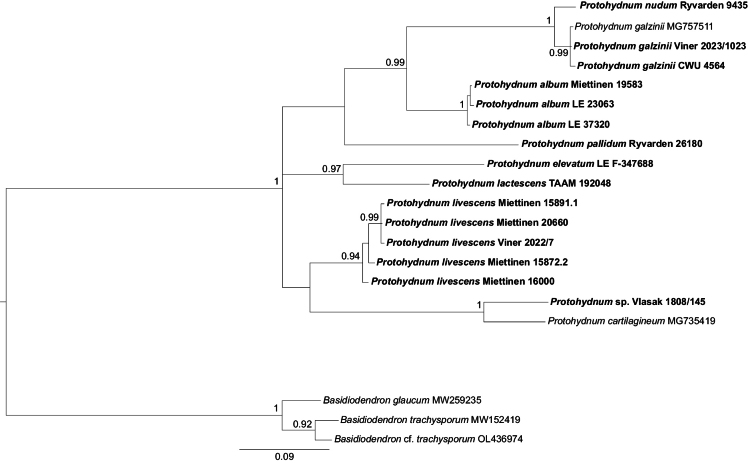
Bayesian majority-rule consensus ITS-based tree of the Protohydnum clade. Support values are given as Bayesian posterior probabilities. *Basidiodendron* spp. were used as an outgroup.

**Figure 3.**
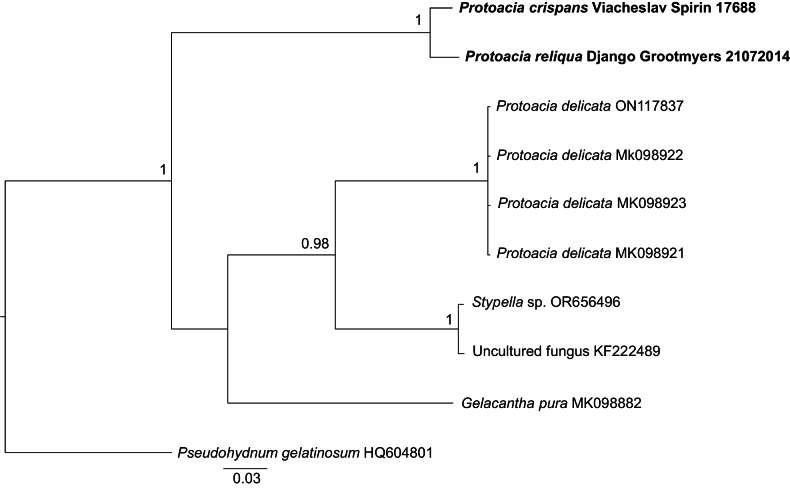
Bayesian majority-rule consensus ITS-based tree of the Protoacia – Gelacantha clade. Support values are given as Bayesian posterior probabilities. *Pseudohydnumgelatinosum* (Scop.) P. Karst. was used as an outgroup.

**Figure 4.**
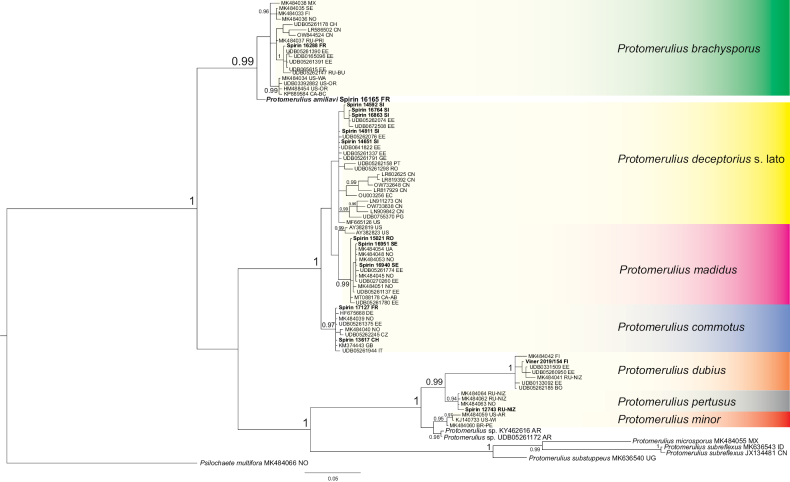
Bayesian majority-rule consensus ITS-based tree of the Protomerulius clade. Support values are given as Bayesian posterior probabilities. *Psilochaetemultifora* Spirin & Malysheva was used as an outgroup.

**Figure 5.**
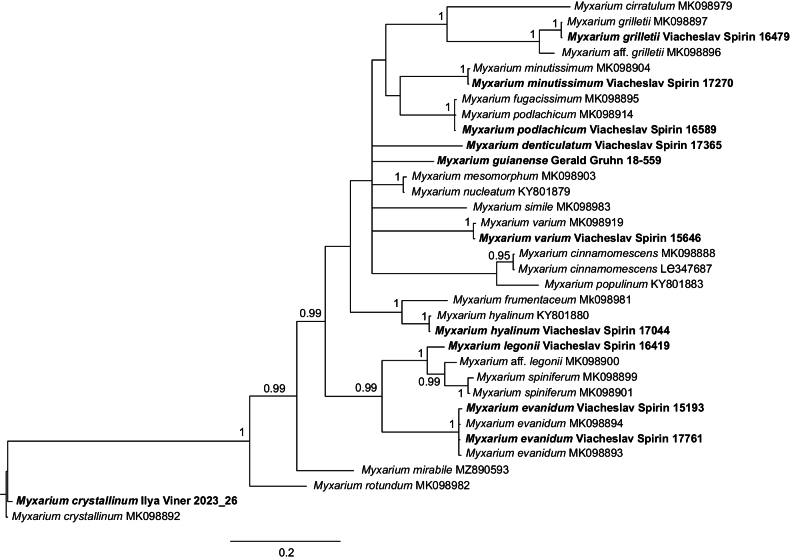
Bayesian majority-rule consensus ITS-based tree of the Myxarium clade. Support values are given as Bayesian posterior probabilities. *Myxariumcrystallinum* was used as an outgroup.

The newly generated sequences were compiled into five sequence datasets, which were complemented with relevant sequences from the literature. For each dataset, BLAST searches in GenBank and UNITE were used to locate highly similar public sequences, primarily of the environmental sequencing type, which, if found, were added to the respective dataset. Each dataset was aligned using MAFFT v. 7.520 (Rozewicki et al. 2019) and adjusted manually. Most identical and near-identical sequences were deleted to minimise redundancy. Five sets of phylogenetic analyses were performed: for Fig. 1, both Bayesian inference and maximum-likelihood analysis were undertaken. For simplified versions of Figs 2–5, only Bayesian inference was undertaken. MrModelTest v. 2.4 (Nylander 2004) was used to select the model of nucleotide evolution for the Bayesian analysis. As applicable, models were estimated separately for ITS1, 5.8S, ITS2, and LSU. The Bayesian analysis was carried out in MrBayes 3.2.7a (Ronquist et al. 2012). Eight MCMCMC chains were run for 10 million generations with a burn-in of 50%. IQ-TREE v. 2.2.2.6 (Minh et al. 2020) was used to infer the maximum likelihood tree in Fig. 1. IQ-TREE was set to estimate the model of nucleotide evolution and was run using default settings. Ten thousand replicates of ultrafast bootstrap (Hoang et al. 2018) were run to estimate clade support.

Sequenced specimens below are marked by an asterisk (*).

## ﻿Results

Five datasets were compiled to reconstruct a genus-level topology of the Auriculariales, with special focus on taxa with stalked basidia (ITS–LSU dataset), and to assess species diversity in selected genera (four ITS datasets). The multiple sequence alignments – annotated with the number of taxa, characters, models of nucleotide evolution, and details of the corresponding *MrBayes* run – are available at https://doi.org/10.15156/BIO/3301247.

The ITS–LSU dataset encompasses 65 species of the Auriculariales and six species as an outgroup (five members of the Sebacinales M. Weiß, Selosse, Rexer, A. Urb. & Oberw., plus *Exidiopsisgloeophora* (Oberw.) Wojewoda as *incertae sedis*). The Auriculariales with stalked basidia are distributed among fifteen clades/single-species lineages. Of these, *Elmerina* Bres. (incl. *Protodaedalea* Imazeki), *Protodontia* Höhn., *Myxariellum* Spirin & Malysheva, *Pseudohydnum*, *Hyalodon* Malysheva & Spirin, *Stypellopsis* Spirin & Malysheva, and *Ofella* Spirin & Malysheva have been investigated in previous publications (Sotome et al. 2014; Malysheva et al. 2018; Spirin et al. 2018a, 2019a, b, c, 2023; Chen et al. 2020; Zhou et al. 2022, 2023; Coelho-Nascimento et al. 2024), while *Tremiscus* (Pers.) Lév. still awaits a proper revision. In the present study, we focus on the following clades in the tree (Fig. 1):

1. The *Protohydnum* clade (pp = 0.99, bs = 90%). As a monophyletic lineage, this group was first recognised by Weiß and Oberwinkler (2001), who detected *Bourdotiagalzinii* (Bres.) Torrend (the generic type of *Bourdotia* (Bres.) Bres. & Torrend), *Ductiferapululahuana* (Pat.) Donk (senior synonym of *Ductiferamillei* Lloyd, the generic type of *Ductifera* Lloyd), and *Ductiferasucina* (Möller) K. Wells in a strongly supported clade. They stressed clear morphological similarities between *Bourdotia* and *Ductifera* (gelatinous basidiocarps, presence of gloeocystidia) and suggested that they should be merged. Malysheva et al. (2018) found that the generic type of *Protohydnum*, *P.cartilagineum* Möller, is a member of the *Bourdotia*–*Ductifera* clade as well, which was again strongly supported. From a morphological perspective, including *P.cartilagineum*, an acystidiate, strongly hydnoid species, in one genus with *Bourdotia* and *Ductifera* appeared somewhat problematic. In the present study, we expand sampling of this clade with six additional species, of which four are newly described, one is reassessed (*Exidiopsislivescens* (Bres.) Bourdot & Maire) and given a new combination, and one is left unnamed due to the lack of fertile material.

Our current data indicate that the presence of gloeocystidia cannot be considered a reliable character for the clade, as five of the ten species included in the ITS–LSU phylogeny lack them. These acystidiate species (including the type species of *Protohydnum*) cluster together (pp = 1, bs = 100%) and might be taxonomically interpreted as belonging to one well-defined genus (*i.e.*, *Protohydnum* s. str.). However, separating them from the rest of the taxa of the broader *Protohydnum* clade (*i.e.*, *Bourdotia* and *Ductifera* spp.) would necessitate the introduction of three more genera, none of which would have distinct morphological differences. In particular, all species of *Protohydnum* s. str. possess stalked basidia with a stipe gradually widening towards the apical cells, the so-called petiolate basidia (cf. Wells and Raitviir 1975). Basidia of the same kind are characteristic of resupinate species in three other subclades of the *Protohydnum* clade, including *Bourdotiagalzinii* (see Fig. 1), while species with cerebriform or cushion-shaped basidiocarps (*Ductifera* spp.) have sessile basidia (*i.e.*, four-celled basidia devoid of a stalk). However, two of the three subclades contain both resupinate species with petiolate basidia and cerebriform species with sessile basidia, and it is therefore impossible to characterise them based on these traits. Other morphological characters (*e.g.*, basidiospore shape and size) also do not provide significant grounds for defining these subclades. For this reason, we consider maintaining *Protohydnum*, *Bourdotia*, and *Ductifera* spp. in one genus as the most reasonable solution with our current knowledge of this group. A newly described *P.nudum* is included in the ITS dataset (Fig. 2). Additionally, five more species are recombined below to *Protohydnum* based on morphological evidence following examination of their types.

2. The *Elmericium* lineage in the *Elmerina* clade (pp = 0.95, bs = 82%, Fig. 1) is represented by the single newly described species *Elmericiumalabastrinum*. Unlike other members of the clade, of which all but one are poroid, *E.alabastrinum* has corticioid, gelatinous basidiocarps. Morphologically and phylogenetically, we believe it is sufficiently distant from *Elmerina* and its relatives to warrant placement in a genus of its own.

3. The *Hydrophana* clade (pp = 1, bs = 100%, Fig. 1). The genus *Hydrophana* Malysheva & Spirin was recently introduced as a result of rearranging the *Myxarium*-like species, and it was, until now, limited to one species, *Hydrophanasphaerospora* (Bourdot & Galzin) Malysheva & Spirin (Spirin et al. 2019b). In the present study, we add two new species to the genus, viz., *Hydrophanafessula* from northern Europe and *Hydrophanatrichiesiana* from South America. We amend the generic description to encompass species with both ellipsoid and broadly cylindrical basidiospores.

4. The *Mycostilla* clade (pp = 1, bs = 100%, Fig. 1). The genus *Mycostilla* Spirin & Malysheva was originally described as monotypic, with *Mycostillavermiformis* (Berk. & Broome) Spirin & Malysheva as its only species (Spirin et al. 2019a). Here, we introduce a new species in the genus, *Mycostillachromatica*. *M.chromatica* is macroscopically quite different from *M.vermiformis*, and it lacks the cystidia that are characteristic of the latter species. We therefore amend the generic description of *Mycostilla* below. The two *Mycostilla* species were recovered as part of a larger, strongly supported clade that also includes the generic type of *Endoperplexa* P. Roberts, *Endoperplexadartmorica* P. Roberts (pp = 1, bs = 100%, Fig. 1). At present, we refrain from merging these two genera, mainly because of the rather distinct morphology of *E.dartmorica* (species with constantly sessile basidia) and the notable phylogenetic distance between it and *Mycostilla* spp. Other *Endoperplexa* species are not closely related to *E.dartmorica* and *Mycostilla* spp. (unpublished data).

5. The *Gelacantha*–*Protoacia* clade (pp = 0.95, bs = 97%, Fig. 1). The type species of two recently described genera, *Gelacanthapura* Malysheva & Spirin and *Protoaciadelicata* Spirin & Malysheva, cluster together in the ITS–LSU tree, although the clade gains sufficient support only in the ML phylogeny. With our current knowledge of the group, we prefer to keep these genera separate due to differing basidiocarp morphology and highly divergent ITS sequences. A new species, *Protoaciareliqua* from North America, is introduced and assigned to *Protoacia*, primarily because of a higher morphological similarity with *P.delicata* than with *G.pura*. However, its phylogenetic position could not be confidently resolved, as it is equally distant from the type species of both genera. One more new species, *Protoaciacrispans* from Europe, is described as closely related to *P.reliqua*, and a higher species diversity in this group is suggested based on environmental ITS sequences (Fig. 3). Nonetheless, any definitive conclusion about generic division in this clade (one large genus versus three genera – *i.e.*, separate *Gelacantha* Malysheva & Spirin and *Protoacia* Spirin & Malysheva, plus a new genus for *P.reliqua* and *P.crispans*) would be premature without broader taxon sampling and additional genetic markers.

6. The *Protomerulius* clade (pp = 1, bs = 100%, Fig. 1). In its current scope, *Protomerulius* Möller embraces mainly corticioid species formerly considered members of *Heterochaetella* (Bourdot) Bourdot & Galzin, while poroid genera originally assigned to the genus represent a minority (Spirin et al. 2019c). Here, we introduce two more corticioid species from Europe, *Protomeruliusamiliavi* and *Protomeruliusdeceptorius*, and expand our current knowledge of *Protomerulius* species diversity and distribution using available environmental data (Fig. 4).

7. The *Myxarium* clade (pp = 1, bs = 100%, Fig. 1). The genus *Myxarium* Wallr. was recently reassessed through combined morphological and phylogenetic evidence. It is recovered as a strongly supported clade in our ITS–LSU tree. Three new *Myxarium* species are described below, viz. *Myxariumguianense* (Fig. 1), *Myxariumdenticulatum*, and *Myxariumspiniferum* (Fig. 5). The identity of *M.legonii* (P. Roberts) P. Roberts is reconsidered based on new morphological and DNA data, and *Tremellainconspicua* Pat. is formally transferred to *Myxarium* based on a morphological study of its type.

## ﻿Taxonomy

### ﻿ Elmericium

Taxon classificationFungiAuriculariales

Spirin & V. Malysheva gen. nov.

496E9FBA-81DB-524A-9690-3734A15C5B62

858658

#### Etymology.

Elmericium – derived from *Elmerina*, a phylogenetically close and anatomically similar genus, and *Corticium*, in reference to the crust-like basidiocarps.

#### Description.

Basidiocarps effused, smooth or nearly so, gelatinous, opalescent or opaque, light-coloured, margin sharply delimited. Hyphal structure monomitic, hyphae hyaline or brownish, clamped. Cystidia present, hyaline, thin-walled, clavate to somewhat tapering. Hyphidia abundant, richly branched, forming a continuous layer. Basidia four-celled, longitudinally septate, ovoid-ellipsoid to obconical, petiolate, embedded. Basidiospores smooth, thin-walled, broadly ellipsoid to ellipsoid. On dead wood of deciduous trees.

#### Type species.

*Elmericiumalabastrinum*.

### ﻿ Elmericium alabastrinum

Taxon classificationFungiAuriculariales

Spirin & V. Malysheva sp. nov.

D5449F1A-283E-5BF2-A020-36A6289D9AC3

858659

Figs 7A , 8A

#### Holotype.

Russia. Khabarovsk Reg.: Khabarovsk Dist., Hologu, *Populusmaximowiczii* (partly corticated fallen log), 17.VIII.2012 Spirin 5311* (H, isotype – in LE).

#### Etymology.

Alabastrinus (Lat., adj.) – alabaster-like; in reference to the hymenium colours.

#### Description.

Basidiocarps effused, up to 5 cm in widest dimension, smooth or indistinctly tuberculate, gelatinous, opalescent, greyish-white, often with reddish-brown spots, 0.5–1 mm thick, in dry condition opaque, pinkish-grey or grey, with vinaceous-brown or brownish-black stains, crustose, margin sharply delimited, usually slightly elevated, adnate or detaching, concolourous with hymenial surface. Hyphal structure monomitic, hyphae hyaline or brownish, clamped; subicular hyphae thick-walled, subparallel, 3–5 μm in diam., subhymenial hyphae thin- to slightly thick-walled, interwoven or ascending, densely arranged, 2–4 μm in diam., with occasional inflations up to 6 μm in diam. Cystidia abundant, hyaline, clavate to somewhat tapering, 20–30 × 5–8 μm, slightly projecting. Hyphidia abundant, richly branched, 1–2 μm in diam. at the apex, usually forming a continuous layer up to 15 μm thick. Basidia four-celled, longitudinally septate, ovoid-ellipsoid to obconical, pedunculate (petiolate), (16–) 17–27.5 (–30) × (9.8–) 10.1–14.0 (–14.3) μm (n = 30/2), stalk up to 26 × 5–6 μm, narrowing to the base, sterigmata tubular, gradually tapering, up to 23 × 3–4 μm. Basidiospores smooth, thin-walled, broadly ellipsoid to ellipsoid, (8.0–) 8.6–12.2 (–12.3) × (5.7–) 5.9–8.1 (–8.2) μm (n = 50/2), L = 10.23–10.32, W = 7.03–7.20, Q’ = (1.2–) 1.3–1.7 (–1.8), Q = 1.43–1.48.

#### Distribution and ecology.

East Asia (Russian Far East); corticated logs or still-attached branches of deciduous trees (mostly *Populus*) along riversides.

#### Remarks.

At present, *Elmericiumalabastrinum* is the single known corticioid representative of the *Elmerina* clade in the Auriculariaceae Fr. ex Lindau. All other members of this group (*Aporpium* Bondartsev & Singer ex Singer, *Elmerina*, and *Protodaedalea*) are poroid except *Elmerinasclerodontia* (Mont. & Berk.) Miettinen & Spirin, which has clavarioid basidiocarps (see Malysheva et al. 2018). *Elmericiumalabastrinum* has petiolate basidia nearly identical in shape and size to those of *Elmerina* and *Protodaedalea*. However, the rest of the macroscopic traits (smooth, crust-like, gelatinous basidiocarps vs. poroid, cartilagineous, or leathery ones) and anatomical features (hyphal structure, hymenial construction, and basidiospore shape) clearly separate *Elmericium* from its relatives. The freshly collected basidiocarps of *E.alabastrinum* are usually sterile, although they start active sporulation after being rehydrated for several hours.

### Hydrophana

Taxon classificationFungiAuriculariales

V. Malysheva & Spirin, Nordic Journal of Botany 37 (e02394): 8, 2019, emend.

2A23DBE0-06C4-5B37-8C91-50789EB51BB8

#### Description.

Basidiocarps effused, continuous, smooth or tuberculate, gelatinous, thin. Hyphal structure monomitic; hyphae clamped. Cystidia absent; hyphidia present, richly branched, 0.5–1.5 μm in diam. Basidia four-celled, longitudinally septate, broadly ellipsoid to globose, pedunculate (stalk occasionally reduced). Basidiospores hyaline, thin-walled, broadly cylindrical or broadly ellipsoid to globose. On rotten wood.

#### Type species.

*Sebacinasphaerospora* Bourdot & Galzin.

Originally, *Hydrophana* was introduced to encompass one species, *H.sphaerospora* (Bourdot & Galzin) Malysheva & Spirin (formerly *Myxariumsphaerosporum* (Bourdot & Galzin) D.A. Reid), which turned out to be not closely related to the rest of the *Myxarium*-like taxa. Morphologically, *Hydrophana* was distinguished from *Myxarium* s. lato mainly due to broadly ellipsoid or subglobose basidiospores in the type species (Spirin et al. 2019b). However, adding two new *Hydrophana* species with broadly cylindrical or ellipsoid basidiospores necessitates a redefinition of the genus. In its current scope, *Hydrophana* can be separated from the ellipsoid-spored *Myxarium* spp. due to continuous (not reticulate or pustulate-coalescing) basidiocarps, smooth (or nearly so) hymenophore, narrower hyphidia, and slenderer sterigmata. The two latter features can be used for distinguishing *Hydrophana* spp. from *Ofellaglaira* (Lloyd) Spirin & Malysheva. In turn, *Myxariellum* spp. have well-differentiated tapering cystidia, and their basidia possess a thicker stalk and wider sterigmata than do *Hydrophana*.

### ﻿ Hydrophana fessula

Taxon classificationFungiAuriculariales

Viner & Spirin sp. nov.

2AC14923-B74C-5E4A-9ABC-939BE380EDA5

858660

Fig. 8B

#### Holotype.

Finland. Uusimaa: Helsinki, Talosaari, *Piceaabies* (fallen decorticated log), 13.X.2021 Viner 2021/289* (H 6112924).

#### Etymology.

Fessulus (Lat., adj.) – limp; in reference to quickly collapsing hyphae.

#### Description.

Basidiocarps effused, up to 4 cm in widest dimension, smooth or tuberculate, gelatinous, semitranslucent, greyish, 0.4–0.7 mm thick, in dry condition vernicose, hardly visible, margin gradually thinning-out. Hyphal structure monomitic, hyphae hyaline, clamped; subicular hyphae with a distinct wall, interwoven, 2–4 μm in diam., subhymenial hyphae thin-walled, quickly collapsing, ascending or interwoven, 2–3 μm in diam. Cystidia absent. Hyphidia abundant, richly branched, 0.5–1 μm in diam. at the apex, partly covering hymenial cells. Basidia four-celled, longitudinally septate, broadly ellipsoid to globose, pedunculate, (8.7–) 8.8–10.2 (–10.8) × (7.2–) 7.3–8.7 (–9.0) μm (n = 20/1), stalk distinct, up to 30 × 1.5–2.5 μm, sterigmata gradually tapering, occasionally bifurcate, up to 25 × 1.8–2.2 μm. Basidiospores smooth, thin-walled, broadly cylindrical to broadly ellipsoid, more rarely lacrymoid or ovoid, (5.2–) 5.3–7.2 × (3.8–) 4.0–5.2 (–5.4) μm (n = 30/1), L = 6.31, W = 4.58, Q’ = (1.2–) 1.3–1.6 (–1.7), Q = 1.39, often with a large central oil drop.

#### Distribution and ecology.

Europe (Finland); decorticated coniferous logs (*Picea*).

#### Remarks.

*Hydrophanafessula* is described here as the second European representative of the genus. It differs from *H.sphaerospora* mainly in having broadly cylindrical/ellipsoid basidiospores (broadly ellipsoid to nearly globose in the latter species). Moreover, *H.fessula* was found on coniferous wood, while *H.sphaerospora* seems to be restricted to deciduous trees. *Ofellaglaira*, also inhabiting coniferous hosts, can easily be mistaken for *H.fessula*. However, it has thinner basidiocarps as well as wider hyphidia and sterigmata, and it occasionally produces hymenial cystidia. Basidiospores of *O.glaira* are more regular in shape than in *H.fessula*, varying from ellipsoid to subglobose (see description in Spirin et al. 2019b). Both species seem to be very rare. While *H.fessula* is currently known from the type locality only, *O.glaira* has been detected a few times in Estonia, Finland, Norway, and Sweden (the *locus classicus*). Additionally, the GenBank sequence HQ441914 of an uncultured fungus isolate from Finland (Rajala et al. 2011) belongs to *O.glaira*.

### ﻿ Hydrophana trichiesiana

Taxon classificationFungiAuriculariales

Gruhn & Rödel sp. nov.

D3D2B530-A258-557F-A9DB-B0AB4EE99D87

858661

Figs 6A , 7B , 8C

#### Holotype.

French Guiana. Régina: Noruragues, Saut Pararé, rotten wood of angiosperm, 4.XII.2018 Gruhn GUY18-573* (LIP, isotype – H).

#### Etymology.

Trichiesianus (Lat., adj.) – in homage to Gérard Trichies, a famous discoverer of minuscule heterobasidiomycetes.

#### Description.

Basidiocarps effused, up to 3 cm in widest dimension, tuberculate, gelatinous, opalescent, cream-coloured to pale ochraceous, 0.2–0.3 mm thick, in dry condition ochraceous-brown, vernicose, margin gradually thinning-out. Hyphal structure monomitic, hyphae hyaline, clamped; subicular hyphae thin-walled, interwoven or subparallel, 2–3 μm in diam., subhymenial hyphae very thin-walled, quickly collapsing, interwoven, rather densely arranged and partly glued together, 1–2 (–2.5) μm in diam. Cystidia absent. Hyphidia abundant, richly branched, 0.8–1.2 μm in diam. at the apex, partly covering hymenial cells. Basidia four-celled, longitudinally septate, broadly ellipsoid to globose, sessile or pedunculate, (8.0–) 8.2–9.8 (–10.1) × (7.4–) 7.8–8.8 (–9.0) μm (n = 20/1), stalk usually strongly reduced, up to 3 × 2 μm, sterigmata gradually tapering, up to 18 × 1.5–2 μm. Basidiospores smooth, thin-walled, ellipsoid to broadly ellipsoid, the longest spores broadly cylindrical and sometimes slightly curved, (5.1–) 5.2–7.0 (–7.2) × (3.7–) 3.8–4.7 (–4.8) μm (n = 30/1), L = 6.08, W = 4.21, Q’ = (1.2–) 1.3–1.7 (–1.8), Q = 1.45, often with a large central oil drop.

**Figure 6.**
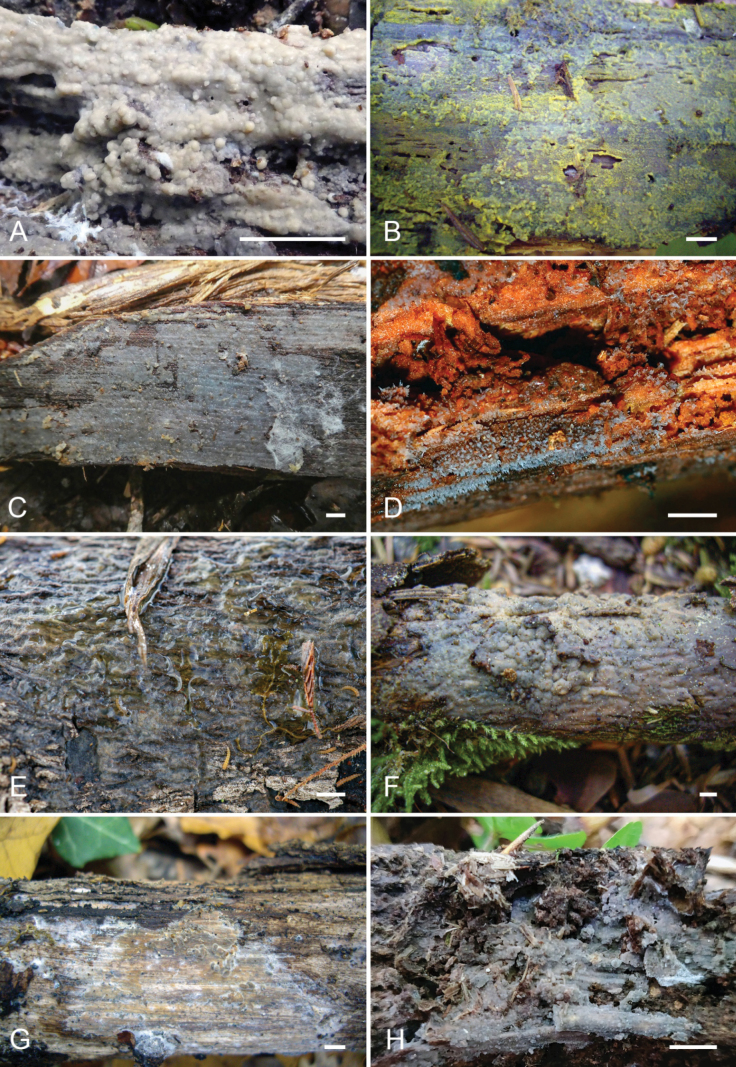
Basidiocarps of: **A.***Hydrophanatrichiesiana* (holotype); **B.***Mycostillachromatica* (holotype); **C.***Myxariumguianense* (holotype); **D.***Protoaciareliqua* (holotype); **E.***Protohydnumerumpens* (holotype); **F.***P.livescens* (specimen Spirin 15619); **G.***Protomeruliusamiliavi* (holotype); **H.***P.deceptorius* (specimen Spirin 16784). Scale bar: 1 cm.

#### Distribution and ecology.

South America (French Guiana); decorticated, decayed angiosperm wood.

**Figure 7.**
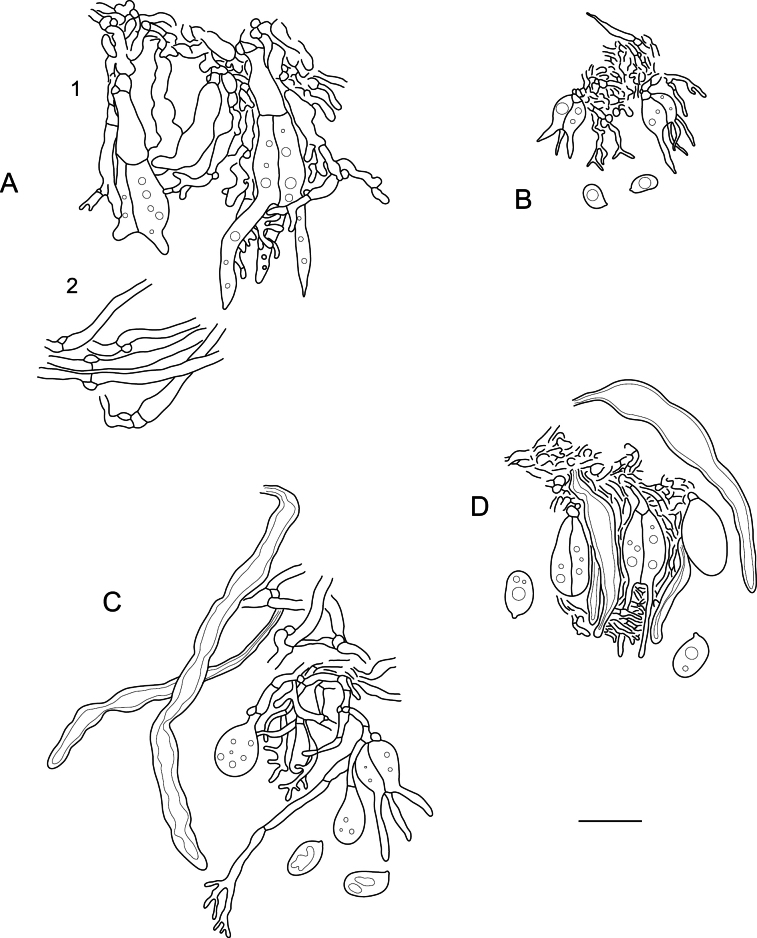
Microscopic structures of: **A.***Elmericiumalabastrinum* (holotype) (1 – hymenial cells and subhymenial hyphae; 2 – subicular hyphae); **B.***Hydrophanatrichiesiana* (holotype); **C.***Protohydnumalbum* (specimen Miettinen 196583); **D.***P.galzinii* (specimen Miettinen 15900.4). Scale bar: 10 µm.

#### Remarks.

*Hydrophanatrichiesiana* is described here as the first representative of the genus found in the tropics. It differs from the two other species of the genus, *H.fessula* and *H.sphaerospora*, in having basidia with a strongly reduced, although still detectable, stalk. Basidiospores of *H.trichiesiana* are similar to those of *H.fessula*, although slightly narrower on average.

**Figure 8.**
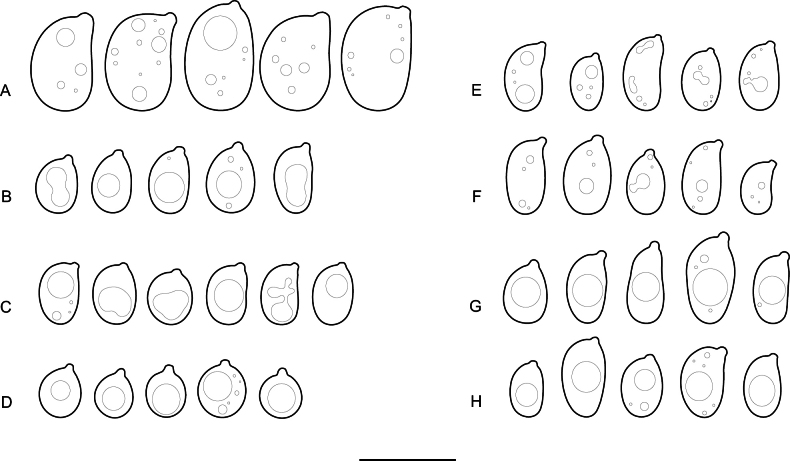
Basidiospores of: **A.***Elmericiumalabastrinum* (holotype); **B.***Hydrophanafessula* (holotype); **C.***H.trichiesiana* (holotype); **D.***Mycostillachromatica* (holotype); **E.***M.guianense* (holotype); **F.***M.inconspicuum* (holotype); **G.***Protoaciacrispans* (holotype); **H.***P.reliqua* (holotype). Scale bar: 10 µm.

### ﻿ Mycostilla

Taxon classificationFungiAuriculariales

Spirin & V. Malysheva, Antonie van Leeuwenhoek 112: 760, 2018, emend.

83D9FC38-2B00-5F48-99C1-B1C5B4ECEA15

#### Description.

Basidiocarps appearing as small gelatinous outgrowths on a hardly visible joint subiculum, later fusing into reticulate or continuous compound fructifications. Hyphal structure monomitic, hyphae clamped. Tramal cystidia tubular, slightly tapering upwards, apically blunt, or absent. Gloeocystidia (if present) running more or less parallel to tramal cystidia. Basidia two – four-celled, pedunculate, with slender, distantly located sterigmata. Basidiospores thin-walled, subglobose, repetitive, often with one large oil drop.

#### Type species.

*Dacrymycesvermiformis* Berk. & Broome.

The generic description of *Mycostilla* is amended here to encompass a new species, *M.chromatica*. In contrast to the generic type, *M.vermiformis*, it has continuous (not reticulate) basidiocarps and lacks cystidia. Nevertheless, it is phylogenetically quite close to *M.vermiformis* and is therefore described as a *Mycostilla*. Basidia of both species are of the same shape (slightly larger in *M.chromatica* than in *M.vermiformis*), and basidiospores are nearly identical.

### ﻿ Mycostilla chromatica

Taxon classificationFungiAuriculariales

Spirin & Grebenc sp. nov.

F29959B6-1F08-5E35-8F32-6EE57FE01EDD

858662

Figs 6B , 8D

#### Holotype.

Slovenia. Kočevje: Podstenice, Rajhenavski Rog, *Abiesalba* (fallen decorticated log), 21.VIII.2021 Spirin 14839* (H, isotype – LJF).

#### Etymology.

Chromaticus (Lat., adj.) – chrome-yellow, in reference to the basidiocarp’s colour.

#### Description.

Basidiocarps effused, first small, then fusing together and reaching up to 20 cm in longest dimension, indistinctly or clearly tuberculate, gelatinous, semitranslucent, containing numerous chrome-yellow grains, 0.1–0.4 mm thick, drying to a bright-yellow thin crust, margin gradually thinning out. Hyphal structure monomitic, hyphae hyaline, clamped, richly encrusted by chrome-yellow angular crystals occasionally fusing together in large amorphous concretions; subicular hyphae thin-walled or with a distinct wall, interwoven, anastomosing, 2–4 μm in diam. (occasionally inflated up to 6 μm in diam.), subhymenial hyphae thin-walled, quickly collapsing, interwoven, 2–3 μm in diam. Cystidia absent. Hyphidia scattered, simple or sparsely branched, 1–1.5 μm in diam. at the apex. Basidia four-celled, longitudinally septate, broadly ellipsoid to globose, pedunculate, (8.3–) 8.7–11.0 (–11.2) × (7.8–) 8.0–9.0 (–9.2) μm (n = 20/2), stalk usually distinct, up to 15 × 2–3 μm, sterigmata gradually tapering, up to 20 × 1.5–2 μm. Basidiospores smooth, thin-walled, ellipsoid to broadly ellipsoid or subglobose, more rarely globose, (4.2–) 4.7–6.1 (–6.2) × (3.6–) 3.8–5.2 (–5.4) μm (n = 60/2), L = 5.20–5.31, W = 4.46–4.78, Q’ = (1.0–) 1.1–1.3 (–1.4), Q = 1.09–1.20, often with a large central oil drop.

#### Distribution and ecology.

Europe (Slovenia); fallen decorticated logs of conifers (*Abies*).

#### Remarks.

*Mycostillachromatica* is a distinctive species due to its brightly coloured, effused, and gelatinous basidiocarps. It has been found twice in the pristine fir-beech forests of Slovenia. The colours of *M.chromatica* are strongly reminiscent of the corticioid fungus *Flavophlebiasulfureoisabellina* (Litsch.) K.H. Larss. & Hjortstam (Agaricales Underw., Basidiomycota). The latter species also occurs on *Abiesalba* logs in old-growth forests of Central Europe. However, it has thicker basidiocarps than *M.chromatica* and is microscopically completely different.

### Myxarium

Taxon classificationFungiAuriculariales

Wallr., Flora Cryptogamica Germaniae 2: 260, 1833.

0AB6EF1F-AD63-542B-8FB3-1D233F518E4A

#### Note.

For a modern description of the genus, see Spirin et al. (2019b).

#### Type species.

*Myxariumnucleatum* Wallr.

### ﻿ Myxarium denticulatum

Taxon classificationFungiAuriculariales

Spirin sp. nov.

CA437C0D-1017-5983-A224-0B86890D45C2

858663

Fig. 9C

#### Holotype.

Sweden. Bohuslän: Hönö, Ersdalsvägen, *Sorbus* sp. (recently fallen corticated branch), 12.VIII.2024 Spirin 17365* (GB, isotype – H).

#### Etymology.

Denticulatus (Lat., adj.) – possessing small teeth.

#### Description.

Basidiocarps effused, small and inconspicuous, up to 8 mm in widest dimension, semitranslucent, gelatinous, greyish, adnate, almost invisible in dry condition; hymenophore hydnoid, spines rather regularly arranged, acute, single, up to 0.1 mm long, 5–6 per mm; subiculum watery greyish, semitranslucent, 0.02–0.03 mm thick; margin gradually thinning-out. Hyphal structure monomitic, hyphae hyaline, clamped; subicular hyphae thin-walled, subparallel, hardly discernible, 1–2 μm in diam., subhymenial hyphae ascending or interwoven, thin-walled, and quickly collapsing, (1.0–) 1.1–2.5 (–2.6) μm in diam. (n = 20/1). Crystals absent. Hyphidia abundant, richly branched, 1–1.5 μm in diam. in the apical part, distributed among basidia and partly covering basidial cells. Basidia two – four-celled, longitudinally septate, broadly ellipsoid to subglobose, pedunculate, (6.5–) 7.0–8.6 (–8.9) × (5.8–) 6.0–7.5 (–7.8) μm (n = 20/1), scattered, stalk up to 15 × 1.5–2 μm, sterigmata up to 10 × 2–2.2 μm, sometimes bifurcate. Basidiospores narrowly ellipsoid to broadly cylindrical, occasionally slightly concave on the ventral side, (5.0–) 5.1–7.0 (–7.3) × 3.0–4.2 (–4.8) μm (n = 33/1), L = 6.18, W = 3.57, Q’ = (1.2–) 1.4–2.0 (–2.1), Q = 1.75, usually with a large central oil drop.

**Figure 9.**
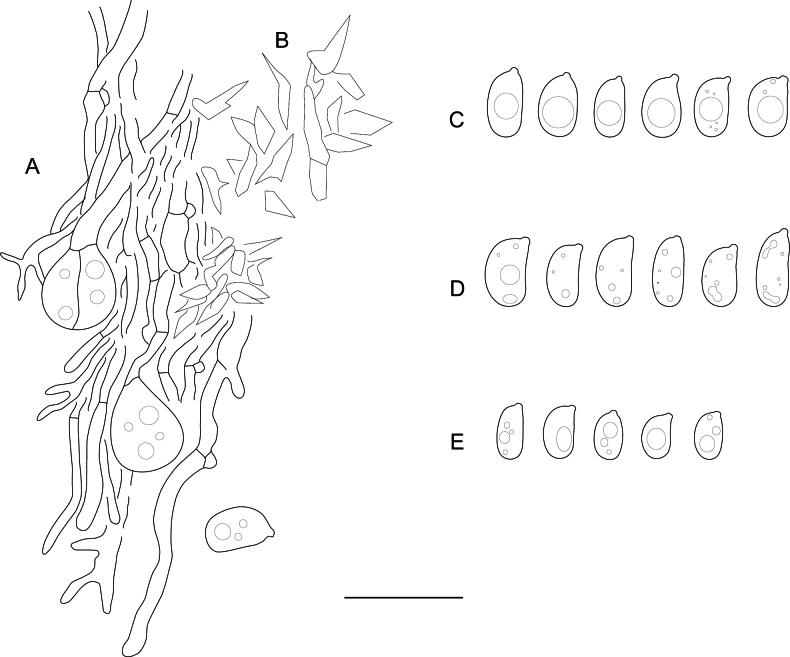
Microscopic structures of the *Myxariumlegonii* complex: **A.***Myxariumlegonii* (specimen Spirin 16419) (hymenial cells and subhymenial hyphae); **B.***M.legonii* (specimen Spirin 16419) (crystals); **C.***M.denticulatum* (holotype) (basidiospores); **D.***M.legonii* (specimen TAAM 132119) (basidiospores); **E.***M.spiniferum* (holotype) (basidiospores). Scale bar: 10 µm.

#### Distribution and ecology.

Europe (Sweden); fallen angiosperm branches (*Sorbus*).

#### Remarks.

*Myxariumdenticulatum* produces extremely thin basidiocarps bearing tiny spines, which are detectable only under magnification. Due to its regularly hydnoid hymenophore, it can be confused with *M.legonii*. However, the latter species bears more pronounced spines and has on average narrower basidiospores (see description below). Another similar species, *Myxariumevanidum* Spirin & K.H. Larss., possesses irregularly arranged spine-like outgrowths on the hymenial surface. These outgrowths disappear completely after drying in *M.evanidum*; in contrast, spines of *M.denticulatum* are visible even in dried material. Under the microscope, *M.denticulatum* and *M.evanidum* are almost indistinguishable. Phylogenetically, *M.denticulatum*, *M.evanidum*, and *M.legonii* are not closely related (Fig. 5). *Myxariumdenticulatum* is currently known only from the type locality, but it is presumably overlooked elsewhere due to its highly diminutive basidiocarps.

### ﻿ Myxarium guianense

Taxon classificationFungiAuriculariales

Gruhn & Spirin sp. nov.

495A73B4-3EAB-5062-BDB9-5B2D9F91AD6C

858664

Figs 6C , 8E

#### Holotype.

French Guiana. Régina: Noruragues, Saut Pararé, decorticated log on the ground, 3.XII.2018 Gruhn GUY18-559* (LIP, isotype – H).

#### Etymology.

Guianensis (Lat., adj.) – originating from French Guiana.

#### Description.

Basidiocarps first appearing as small pustules, 0.03–0.05 mm in diam., then fusing together and forming a compound basidiocarp, up to 3 cm in widest dimension, indistinctly tuberculate, gelatinous, opalescent, greyish, 0.1–0.2 mm thick, in dry condition vernicose, hardly visible, margin sharply delimited, adnate. Hyphal structure monomitic, hyphae hyaline, clamped, frequently anastomosing; subicular hyphae with a distinct wall, interwoven or subparallel, 1.5–5 (6) μm in diam., often slightly inflated at septa, subhymenial hyphae thin-walled or with a distinct wall, tightly glued together, ascending or interwoven, 1–4 μm in diam. Cystidia rare, distinctly tapering to almost subulate, 16–26 × 5.0–8.4 μm (n = 5/1), projecting up to 15 μm above hymenial layer. Hyphidia richly branched, 1–1.5 μm in diam. at the apical part, scattered among basidia. Basidia four-celled, longitudinally septate, ellipsoid to broadly ellipsoid, pedunculate, (7.7–) 7.8–9.4 (–9.7) × (6.5–) 6.6–7.2 (–7.3) μm (n = 20/1), stalk distinct although sometimes strongly reduced, up to 10 × 2–2.5 μm, sterigmata gradually tapering, rarely bifurcate, up to 10 × 1.2–1.5 μm. Basidiospores smooth, thin-walled, narrowly ellipsoid to cylindrical, the longest spores often slightly curved, (5.4–) 5.6–7.3 (–7.8) × (3.0–) 3.1–4.1 (–4.4) μm (n = 36/1), L = 6.65, W = 3.71, Q’ = (1.4–) 1.5–2.1 (–2.3), Q = 1.80.

#### Distribution and ecology.

South America (French Guiana); decorticated angiosperm wood.

#### Remarks.

Morphologically, *M.guianense* is most similar to the European *Myxariumminutissimum* (Höhn.) Spirin & Trichies. However, the initially pustulate basidiocarps of *M.guianensis* fuse together completely and produce a crustaceous continuous one, while in *M.minutissimum* they fuse only partly, and therefore the compound basidiocarps have a very characteristic reticulate shape. Moreover, *M.guianense* differs from *M.minutissimum* in having cystidia. Phylogenetically, these species are not closely related (Figs 1, 5). Differences between *M.guianense* and *M.inconspicuum* are discussed under the latter species. *Myxariumguianense* is so far known from the type locality in French Guiana.

### ﻿ Myxarium inconspicuum

Taxon classificationFungiAuriculariales

(Pat.) Spirin comb. nov.

E4165DD1-FBAE-5B6D-B056-DCEA3A8A44F6

858675

Fig. 8F

≡ Tremellainconspicua Pat., Bulletin de la Société Mycologique de France 9: 138, 1893. Holotype. Ecuador. Pichincha: Quito (surroundings), dead wood, II.1892 Lagerheim (FH00060386, studied).

#### Description.

Basidiocarps first pustular, 0.2–0.4 mm in diam., then fusing together and forming a compound basidiocarp, up to 5 mm in widest dimension, indistinctly tuberculate, gelatinous, opalescent, whitish, 0.1–0.2 mm thick, in dry condition hardly visible, margin sharply delimited, adnate. Hyphal structure monomitic, hyphae hyaline, clamped; subicular hyphae totally collapsed, subhymenial hyphae thin-walled, ascending, 1.5–2.5 μm in diam. Hyphidia abundant, richly branched, 1–2 μm in diam. at the apical part, scattered among basidia and partly covering basidial cells. Basidia four-celled, longitudinally septate, ellipsoid to broadly ellipsoid, pedunculate, (8.1–) 8.3–9.2 (–10.6) × (6.2–) 6.5–8.2 (–8.6) μm (n = 20/1), stalk up to 15 × 2–2.5 μm, sterigmata up to 7 × 1.5 μm. Basidiospores smooth, thin-walled, narrowly ellipsoid to ellipsoid or more rarely cylindrical, (5.0–) 5.7–8.2 (–8.9) × (3.4–) 3.6–5.0 (–5.1) μm (n = 30/1), L = 6.99, W = 4.30, Q’ = (1.3–) 1.4–2.0 (–2.2), Q = 1.63.

#### Remarks.

*Tremellainconspicua* was described from Ecuador (Patouillard and Lagerheim 1893), and its connection with *Myxarium* was discussed by Spirin et al. (2019b). We restudied the type specimen of *T.inconspicua* and are fairly confident that it belongs to *Myxarium*. Both macroscopically and anatomically, *M.inconspicuum* is similar to the European *Myxariumgrilletii* (Boud.) D.A. Reid; it differs from the latter species in having predominantly narrowly ellipsoid spores with a convex ventral side, while in *M.grilletii* (European specimens) the spores are mainly cylindrical or broadly cylindrical and often somewhat curved. However, the North American specimens of *M.grilletii* studied and illustrated by Spirin et al. (2019b) possess predominantly ellipsoid basidiospores. An ITS sequence obtained from the Canadian specimen of *M.grilletii* (GenBank MK098896, designated as Myxariumaff.grilletii in Fig. 5) is identical with an unnamed sequence from the USA (GenBank KX193945), and they clearly deviate from *M.grilletii* sequences from Europe. They may therefore represent a separate species and may in fact be conspecific with *M.inconspicuum*. Answering this question is not feasible without access to newly collected and sequenced material from Ecuador. *Myxariumguianense* introduced above differs from *M.inconspicuum* by its smaller discrete basidiocarps, slightly narrower basidiospores, and the presence of cystidia.

### ﻿ Myxarium legonii

Taxon classificationFungiAuriculariales

(P. Roberts) P. Roberts, Nordic Journal of Botany 37 (e02394): 15, 2019.

0B4CDC59-38C2-5014-B737-D159FF4CC744

Fig. 9A, B, D

≡ Stypellalegonii P. Roberts, Mycotaxon 69: 228, 1998. Holotype. United Kingdom. England: Surrey, Runnymede, Cooper’s Hill, Ulmus sp., 5.III.1988 Legon (K(M) 49367).

#### Description.

Basidiocarps effused, covering a few mm, semitranslucent, gelatinous, whitish or greyish, adnate; hymenophore hydnoid, spines regularly arranged, acute, single or fasciculate, 0.1–0.5 mm long, 5–7 per mm; subiculum first watery greyish, semitranslucent, then whitish, opaque, sometimes shining, 0.02–0.05 mm thick; margin gradually thinning-out. Hyphal structure monomitic, hyphae hyaline, clamped; subicular hyphae thin-walled, predominantly subparallel, 1–3 μm in diam., tramal hyphae subparallel, subhymenial hyphae ascending, very thin-walled, and quickly collapsing, (1.2–) 1.6–2.6 (–3.2) μm in diam. (n = 60/3). Acicular crystals abundant among hyphal tissues, often aggregated in large groups up to 20 μm in the widest dimension. Hyphidia abundant, simple to branched, 1–1.5 μm in diam. at the apical part, distributed among basidia. Basidia four-celled, longitudinally septate, broadly ellipsoid to subglobose, pedunculate, (6.7–) 6.8–9.5 (–9.8) × (5.3–) 5.8–7.3 (–7.4) μm (n = 32/3), scattered, stalk up to 12 × 2–2.5 μm, sterigmata up to 7 × 1.5–2 μm. Basidiospores cylindrical to broadly cylindrical, often slightly curved, (4.6–) 4.9–7.6 (–8.2) × (2.6–) 2.7–4.0 (–4.4) μm (n = 90/3), L = 5.50–6.28, W = 3.29–3.35, Q’ = (1.4–) 1.5–2.3 (–2.6), Q = 1.68–1.89.

#### Distribution and ecology.

Europe (France, Spain, United Kingdom); strongly decomposed wood of angiosperms.

#### Remarks.

Originally described as a member of *Stypella* Möller (Roberts 1998), the species was recently moved to *Myxarium* (Spirin et al. 2019b). The species was, however, accepted as a species complex because available ITS sequences showed clear differences between the European and North American specimens. In the present study, the latter are excluded from our concept of *M.legonii* and assigned to *M.spiniferum*, described below.

Here we reassess *M.legonii* based on newly collected specimens from the western part of Europe, as well as a specimen collected in the *locus classicus*. We found the latter (TAAM 132119) to be morphologically identical to three recent specimens from France and Spain, of which one was sequenced. These all have considerably larger basidiospores than indicated in the protologue (where they were presumably measured from a spore print) and possess abundant acicular crystals in all parts of the basidiocarps (Fig. 9). Phylogenetic analysis shows that the specimen Spirin 9511, treated as *M.legonii* in our earlier study (Spirin et al. 2019b), is not conspecific with the rest of the European collections. This specimen from the European part of Russia has smaller basidia and shorter basidiospores than other European specimens of *M.legonii*, and it lacks crystals. However, all these differences may be age-dependent, and we are therefore unwilling to introduce a new species for this specimen without additional sampling. *Myxariumlegonii* was also reported from Malawi (Spirin et al. 2019b). The identity of this material should be clarified with more specimens from southern Africa.

### ﻿ Myxarium spiniferum

Taxon classificationFungiAuriculariales

Spirin & V. Malysheva sp. nov.

82452FD4-8764-50E9-8EE2-DCB4CF539DE6

858665

Fig. 9E

#### Holotype.

Canada. Alberta: Edmonton, Louise McKinney Riverfront Park, *Populusalba* (rotten decorticated log), 28.VII.2015 Spirin 8986* (H, isotype – LE).

#### Etymology.

Spiniferum (Lat., adj.) – bearing spines.

#### Description.

Basidiocarps effused, up to 3 cm in widest dimension, semitranslucent, gelatinous, whitish or greyish, adnate; hymenophore hydnoid, spines regularly arranged, acute, single or fasciculate, 0.1–0.6 mm long, 5–7 per mm; subiculum first watery greyish, semitranslucent, then whitish, opaque, 0.02–0.05 mm thick; margin gradually thinning-out. Hyphal structure monomitic, hyphae hyaline, clamped; subicular hyphae thin-walled, predominantly subparallel, 1–2 μm in diam., tramal hyphae subparallel, subhymenial hyphae ascending, very thin-walled, and quickly collapsing, (1.6–) 1.9–3.0 (–3.1) μm in diam. (n = 20/1). Acicular crystals quite rare, spread among hyphal tissues, often aggregated in large groups up to 20 μm in the widest dimension. Hyphidia occasionally present, simple to sparsely branched, 1–1.5 μm in diam. at the apical part, distributed among basidia. Basidia four-celled, longitudinally septate, broadly ellipsoid to subglobose, pedunculate, (6.0–) 6.8–8.8 (–9.2) × (5.3–) 5.8–6.9 (–7.0) μm (n = 20/1), partly glued together and often forming a continuous layer, stalk up to 8 × 2 μm, sterigmata up to 10 × 1.8–2.5 μm. Basidiospores cylindrical to broadly cylindrical, occasionally slightly curved, (4.1–) 4.3–5.8 (–5.9) × (2.3–) 2.5–3.7 (–3.8) μm (n = 90/3), L = 5.19–5.27, W = 2.98–3.13, Q’ = (1.4–) 1.5–2.1 (–2.2), Q = 1.70–1.75.

#### Distribution and ecology.

North America (Canada – Alberta, USA – New York, Tennessee); strongly decomposed wood of angiosperms.

#### Remarks.

*Myxariumspiniferum* is described here as the North American sibling species of *M.legonii*. Morphologically, it differs from the latter species in having smaller basidiospores. The photograph of *M.legonii* in Spirin et al. (2019b) belongs to *M.spiniferum*.

### ﻿ Protoacia

Taxon classificationFungiAuriculariales

Spirin & V. Malysheva, Nordic Journal of Botany 37 (e02394): 19, 2019.

D70FAF61-631A-5680-AAF2-240F08191B97

#### Note.

For the genus description, see Spirin et al. (2019b).

#### Type species.

*Protoaciadelicata* Spirin & V. Malysheva.

The generic type of *Protoacia*, *P.delicata*, possesses broadly ellipsoid or subglobose basidiospores. Here we add two new species with ellipsoid to cylindrical basidiospores to *Protoacia*. However, we do not amend the generic description of *Protoacia* due to the somewhat uncertain status of the genus *versus Gelacantha* (see Results). The single species of that genus, *G.pura*, differs from the three *Protoacia* species in having more robust basidiocarps with irregularly distributed and partly fusing spines, as well as wider subicular hyphae and hyphidia. At least one more undescribed species closely related to *P.delicata* can be recognised based on available sequences in GenBank (Fig. 3).

### ﻿ Protoacia crispans

Taxon classificationFungiAuriculariales

Spirin sp. nov.

7E097DA7-C1AE-559C-8E55-AEB9D2CA8E0C

858666

Fig. 8G

#### Holotype.

Sweden. Halland: Särö, Särö Västerskog, *Pinussylvestris* (strongly decayed log with a brown rot), 12.X.2024 Spirin 17688* (GB, isotype – H).

#### Etymology.

Crispans (Lat., present participle of ‘crispo’) – trembling, in reference to delicate consistence of basidiocarps.

#### Description.

Basidiocarps effused, up to 2 cm in widest dimension, semitranslucent, gelatinous, whitish, adnate; hymenophore hydnoid, spines acute, single, 0.1–0.3 mm long, 6–7 per mm; subiculum hardly visible, semitranslucent, 0.02–0.05 mm thick; margin gradually thinning-out. Hyphal structure monomitic, hyphae hyaline, clamped, frequently anastomosing; subicular hyphae thin- to slightly thick-walled, interwoven, 1–3 μm in diam., subhymenial hyphae thin-walled, quickly collapsing, interwoven, 1–3 μm in diam. Cystidia absent. Hyphidia abundant, richly branched, 1–2 μm in diam. at the apex, scattered among basidia or partly covering basidial cells. Basidia (two–) four-celled, longitudinally or obliquely septate, broadly ellipsoid to globose, pedunculate, (9.0–) 9.2–11.9 (–12.0) × (7.2–) 7.3–9.1 (–9.2) μm (n = 20/1), stalk distinct, up to 20 × 2–3.5 μm, sterigmata gradually tapering, rarely bifurcate, up to 15 × 2–3 μm. Basidiospores smooth, thin-walled, narrowly ellipsoid or broadly cylindrical (shorter spores) to cylindrical-subfusiform or somewhat rhomboid (longer spores), more rarely almost pyriform or lacrymoid, (5.2–) 5.9–9.3 (–10.2) × (3.3–) 3.5–5.4 (–5.8) μm (n = 30/1), L = 7.48, W = 4.61, Q’ = (1.3–) 1.4–2.1 (–2.2), Q = 1.64, with a large central oil drop.

#### Distribution and ecology.

Europe (Sweden); very rotten coniferous logs (*Pinus*).

#### Remarks.

*Protoaciacrispans* is morphologically highly similar to *P.delicata*. The two species differ mainly in basidiospore morphology, regularly broadly ellipsoid or subglobose in *P.delicata* and highly diverse, varying from narrowly ellipsoid or broadly cylindrical to subfusiform or lacrymoid in *P.crispans*. Moreover, *P.crispans* has shorter spines than *P.delicata*, which are hardly detectable by the naked eye. Phylogenetically, *P.crispans* is much closer to *P.reliqua* than to *P.delicata*; their differences are listed under the latter species.

*Protodontiasubgelatinosa* (P. Karst.) Pilát is another species that could be mistaken for *P.crispans*. In addition to different substrate preferences (angiosperm wood *versus* conifer wood), *P.subgelatinosa* has wider hyphidia and more regularly shaped, ellipsoid, or ovoid (very rarely cylindrical) basidiospores (see description in Spirin et al. 2019b). Moreover, the spines of *P.subgelatinosa* tend to fuse in groups of three – four, and no such pattern was observed in *P.crispans*.

### ﻿ Protoacia reliqua

Taxon classificationFungiAuriculariales

Grootmyers & Spirin sp. nov.

11B898B4-741C-596C-A3C8-3E4037109AAA

858667

Figs 6D , 8H

#### Holotype.

USA. Tennessee: Anderson Co., Norris, Norris Dam State Park, *Juniperusvirginiana* (fallen decorticated log), 20.VII.2021 Grootmyers 21072014* (H).

#### Etymology.

Reliquus (Lat., adj.) – remaining, another one.

#### Description.

Basidiocarps effused, covering a few cm, semitranslucent, gelatinous, whitish, adnate; hymenophore hydnoid, spines acute, single, 0.05–0.1 mm long, 6–8 per mm; subiculum hardly visible, semitranslucent, 0.02–0.05 mm thick; margin gradually thinning-out. Hyphal structure monomitic, hyphae hyaline, clamped, frequently anastomosing; subicular hyphae thin-walled or with a distinct wall, interwoven, 2.5–4 μm in diam., subhymenial hyphae very thin-walled, quickly collapsing, interwoven, 2–3 μm in diam. Cystidia absent. Hyphidia abundant, richly branched, 1–1.5 μm in diam. at the apex, scattered among basidia. Basidia four-celled, longitudinally septate, broadly ellipsoid to globose, pedunculate, (7.2–) 7.6–9.6 (–10.2) × (5.6–) 6.0–7.3 (–7.5) μm (n = 20/1), stalk distinct, up to 25 × 2–2.5 μm, sterigmata gradually tapering, up to 22 × 2–2.5 μm. Basidiospores smooth, thin-walled, narrowly ellipsoid or ovoid to broadly cylindrical, (5.1–) 5.2–7.7 (–8.1) × (3.2–) 3.3–4.5 (–4.8) μm (n = 31/1), L = 6.20, W = 3.89, Q’ = (1.2–) 1.3–1.8 (–1.9), Q = 1.60, with a large central oil drop.

#### Distribution and ecology.

North America (USA – Tennessee); rotten coniferous logs (*Juniperus* spp.).

#### Remarks.

*Protoaciareliqua* is a North American relative of *P.crispans*. It differs from the latter species in having shorter spines, as well as smaller basidia and basidiospores. The basidiospores of *P.reliqua* are not as variable in shape as those of *P.crispans*, varying from ellipsoid-ovoid to broadly cylindrical. The species is so far known only from the type locality but is likely overlooked due to its diminutive basidiocarps.

### ﻿ Protohydnum

Taxon classificationFungiAuriculariales

Möller, Botanische Mittheilungen aus den Tropen 8: 173, 1895, emend.

86F0FCDE-D8DB-5F31-9294-4514BD496E06

= Bourdotia (Bres.) Bres. & Torrend, Brotéria Serie Botanica 11: 88, 1913. Type species. Sebacinagalzinii Bres. (selected by Clements and Shear 1931: 342).
= Ductifera Lloyd, Mycological Writings 5: 711, 1917. Type species. Ductiferamillei Lloyd.
= Gloeotromera Ervin, Mycologia 48: 692, 1956. Type species. Exidiopsisalba Lloyd.

#### Description.

Basidiocarps cushion-shaped – cerebriform or completely resupinate, with adnate or elevated margin, gelatinous; hymenial surface nearly smooth or (in one species) distinctly hydnoid. Hyphal structure monomitic, hyphae clamped. Gloeocystidia present in most species, tubular, variably tapering upwards, usually embedded. Basidia strictly four-celled, sessile (cerebriform species) or predominantly pedunculate to petiolate (resupinate species), with rather thick, distinctly located sterigmata. Basidiospores thin-walled, cylindrical to ellipsoid, more rarely cylindrical-subfusiform or broadly ellipsoid – subglobose, repetitive.

#### Type species.

*Protohydnumcartilagineum* Möller.

Here we redefine *Protohydnum* and merge it with *Ductifera* and *Bourdotia*. Additionally, two species earlier included in *Exidiopsis* (Bref.) Möller s. lato (i.e., *Exidiopsisglabra* Möller and *E.livescens*) are also reclassified into *Protohydnum*. In its current scope, the genus contains sixteen species.

Phylogenetically, the large *Protohydnum* clade is the sister lineage of *Basidiodendron* Rick (Weiß and Oberwinkler 2001, Spirin et al. 2020, 2021, present study – Fig. 1). All *Basidiodendron* spp. have gloeocystidia similar to those of the cystidiate *Protohydnum* species. However, the genera are quite different otherwise. Basidiocarps of *Basidiodendron* spp. are waxy-arid (not gelatinous as in *Protohydnum*), and they become somewhat gelatinised at the very end of their development in only a few species. Furthermore, turgid basidia of *Basidiodendron* spp. are usually located at the very top of the basidia-bearing hyphae covered by remnants of already collapsed basidial cells, giving these structures a peculiar “fishbone”-like appearance; no such structures have been observed in *Protohydnum* spp. Moreover, basidiospores of *Basidiodendron* spp. feature a large, often asymmetrical, and somewhat eccentric apiculus (at least in the core species of the genus, i.e. those from the *Basidiodendroneyrei* (Wakef.) Luck-Allen and *Basidiodendroncaesiocinereum* (Höhn. & Litsch.) Luck-Allen complexes) (Spirin et al. 2020, 2021). In contrast, the basidiospore apiculus in *Protohydnum* spp. is, as a rule, rather small, regularly outlined, and conventionally located.

### ﻿ Protohydnum album

Taxon classificationFungiAuriculariales

(Lloyd) Spirin comb. nov.

A31B8E47-5927-5541-90FC-E2C82668E799

858676

Figs 7C , 11A

≡ Exidiopsisalba Lloyd, Mycological Writings 4 (44): 9, 1913. Lectotype (selected here, MBT 10025858). Fig. 1929 (plate 177) in Lloyd, Mycological Writings 6, 1921. Epitype (selected here, MBT 10025859). USA. Ohio: Butler Co., Ross Hanover Rd., Platanus sp., 19.VII.1977 Burdsall 9422 (CFMR HHB-9422; duplicate – LE 23063*, studied; other duplicates – ILLS 00157217, MIN 840751, NY 01930202).
= Seismosarcaalba (Lloyd) Lloyd, Mycological Writings 6: 1045, 1921.
= Exidiaalba (Lloyd) Burt, Annals of the Missouri Botanical Garden 8: 366, 1921.
= Gloeotromeraalba (Lloyd) Ervin, Mycologia 48 (5): 692, 1956.

#### Description.

Basidiocarps first orbicular or pulvinate, later cerebriform or foliaceous, gelatinous, opalescent, pure white to beige, later pale ochraceous to brownish, up to 3 cm in diam., 3–15 mm thick, in dry condition brown and tough, margin elevated, partly detaching; lobes rounded, entire, 1–1.5 mm thick. Hyphal structure monomitic, hyphae hyaline, clamped, thin- to slightly thick-walled; context hyphae mostly interwoven, frequently anastomosing, 2–7 μm in diam., occasionally swollen at septa (up to 9 μm in diam.), embedded in gelatinous matrix, subhymenial hyphae predominantly ascending, rather loosely arranged, 1–3 μm in diam. Gloeocystidia abundant, yellowish or brownish, gradually tapering to the apex, more rarely narrowly clavate, occasionally bifurcate, embedded, (69–) 76–260 (–265) × (4.1–) 5.0–11.4 (–13.2) μm (n = 42/4). Hyphidia abundant, richly branched, 0.5–2 μm in diam. at the apex, occasionally forming a continuous layer up to 20 μm thick. Basidia four-celled, longitudinally septate, ovoid-ellipsoid, sessile or very rarely with a strongly reduced stalk-like base (up to 2.5 × 2.5 μm), embedded, (12–) 13–17.5 (–18) × (8.8–) 9.1–13.5 (–13.7) μm (n = 40/4), sterigmata gradually tapering, up to 15 × 2–3 μm. Basidiospores smooth, thin-walled, cylindrical to broadly cylindrical, often slightly curved, (7.3–) 7.6–11.2 (–12.2) × (3.8–) 3.9–6.1 (–6.2) μm (n = 120/4), L = 9.37–10.14, W = 4.63–5.54, Q’ = (1.5–) 1.6–2.3 (–2.4), Q = 1.72–2.04.

#### Distribution and ecology.

North America (USA – the eastern states); wood of angiosperms.

#### Remarks.

The species was first described as *Exidiopsisalba* (Lloyd 1913) and then moved to the genus *Seismosarca* Cooke by Lloyd (1921). Burt (1921) treated *E.alba* under *Exidia*. Martin (1951) studied the original material of *Seismosarcahydrophora* Cooke, the generic type of *Seismosarca*, and concluded that it was an *Auricularia* species. Consequently, Ervin (1956) placed *E.alba*, as well as *Tremellapululahuana* Pat., in a newly established genus, *Gloeotromera* Ervin. Wells (1957) showed that *T.pululahuana* is the same species as *Ductiferamillei*, the generic type of *Ductifera*, which was also described from Pululahua, Ecuador. He therefore moved *T.pululahuana* to *Ductifera* and stated that *E.alba* was conspecific with the former species. We studied both the authentic material of *T.pululahuana* from Ecuador and numerous collections of the species so named from the USA. In our opinion, they should be treated separately, and *E.alba* is to be retained as the correct name for the North American species.

Lloyd did not provide any reference to studied specimens of *E.alba* in the protologue, nor in a later treatment of this species (Lloyd 1913, 1917). Stevenson and Cash (1936) reported 34 specimens in Lloyd’s herbarium labelled as *Heterochaetealba*, *Seismosarcaalba*, or *S.albida*, but none named *E.alba*. Specimens identified as *E.alba* are also lacking in MyCoPortal (Miller and Bates 2017). It is therefore uncertain which collections were at Lloyd’s disposal when he prepared the species description. In the original description, he mentioned that anatomical features of *E.alba* were depicted by E.M. Wakefield (Lloyd 1913: 9). This drawing was published eight years later (Lloyd 1921); we designate it here as a lectotype (iconotype) of *E.alba*. Additionally, we provide *E.alba* with an epitype – a recent sequenced collection distributed among several herbaria.

Sequences of *D.pululahuana* published by Weiß and Oberwinkler (2001) actually belong to *P.album*. Differences between these species are discussed under *Protohydnumpululahuanum*.

### ﻿ Protohydnum aureum

Taxon classificationFungiAuriculariales

(Lowy) Spirin comb. nov.

6FAF8ACE-B0B1-5F40-BC07-5453B0DCE41F

858677

Fig. 11B

≡ Ductiferaaurea Lowy, Mycologia 68: 1106, 1976. Holotype. Panama. Chiriquí: Cerro Punta, Cerro Respingo, unidentified wood in oak forest, 2.VI.1975 Dumont & Carpenter PA-1737 (NY00738326, studied).

#### Description.

Basidiocarps first pustulate, gregarious, up to 1.5 mm in diam., then adpressed-cerebriform, up to 4 mm in diam., finally fusing together and forming compound crust-like fructifications up to 1 cm in the widest dimension, gelatinous, semitranslucent, amber-yellowish to pale ochraceous, 0.5–1 mm thick, in dry condition almost invisible, margin elevated, partly detaching; lobes poorly differentiated, rounded, entire, up to 0.5 mm thick. Hyphal structure monomitic, hyphae clamped; context hyphae thin- to slightly thick-walled, predominantly interwoven, hyaline or brownish, 1–4 μm in diam., embedded in gelatinous matrix, subhymenial hyphae thin-walled, ascending, hyaline, 1.5–3 μm in diam. Gloeocystidia abundant to rather rare, hyaline to yellowish, as a rule gradually tapering to the apex, embedded or rarely slightly projecting, 44–85 × 4.3–8.9 μm (n = 10/1). Hyphidia abundant, simple or sparsely branched, 0.5–1 μm in diam. at the apex, forming a continuous layer up to 10 μm thick. Basidia four-celled, longitudinally septate, ovoid-ellipsoid, sessile, embedded, 18–24 × 9.7–13.5 μm (n = 10/1), sterigmata gradually tapering, up to 15 × 2–3 μm. Basidiospores smooth, thin-walled, cylindrical to broadly cylindrical, occasionally slightly curved, (10.0–) 10.2–14.6 (–15.4) × 5.5–7.2 (–7.4) μm (n = 20/1), L = 12.84, W = 6.46, Q’ = (1.6–) 1.7–2.5 (–2.7), Q = 2.00.

#### Remarks.

*Ductiferaaurea* was described from a single specimen collected in Panama (Lowy 1976) and is so far not known elsewhere. Morphologically, it is most similar to *P.album* and *P.pululahuanum*. It differs from both of these in having considerably larger basidia and basidiospores. We transfer it to *Protohydnum* based on morphological evidence; our attempts to sequence the holotype were unsuccessful.

### Protohydnum cartilagineum

Taxon classificationFungiAuriculariales

Möller, Botanische Mittheilungen aus den Tropen 8: 173, 1895.

4427129F-4DB0-5774-9D72-DF5548BE6EDD

#### Note.

For a modern description of *P.cartilagineum*, see Malysheva et al. (2018). The species is closely related to *P.ocellatum* introduced below; see further remarks under the latter species.

### ﻿ Protohydnum elasticum

Taxon classificationFungiAuriculariales

(Lowy) Spirin comb. nov.

F5C0337F-7ACD-5BD2-9B51-E072BB63FA59

858678

Fig. 11C

≡ Ductiferaelastica Lowy, Mycotaxon 15: 97, 1982. Holotype. Brazil. Acre: Rio Branco, rotten wood, 11.X.1980 Lowy BR646 (LSUM, isotype – NY00738327, studied).

#### Description.

Basidiocarps effused, up to 3 cm in widest dimension, smooth or indistinctly tuberculate, gelatinous, opaque, dirty ochraceous to brownish, 0.1–0.2 mm thick, in dry condition almost invisible, margin sharply delimited, adnate. Hyphal structure monomitic, hyphae hyaline, clamped; subicular hyphae thin- to slightly thick-walled, interwoven or subparallel, hardly discernible, 1.5–3.5 μm in diam., subhymenial hyphae thin-walled, ascending or interwoven, easily collapsing, 1.5–3 μm in diam. Gloeocystidia abundant, hyaline to yellowish, tubular-clavate, sometimes gradually tapering to the apex, embedded, (35–) 36–92 (–102) × (5.0–) 5.1–8.7 (–8.8) μm (n = 14/1). Hyphidia abundant, variably branched, 1–1.5 μm in diam. at the apex, forming a continuous layer up to 10 μm thick. Basidia four-celled, longitudinally septate, ovoid-ellipsoid, pedunculate, 14.5–21 × 9.4–15.2 μm (n = 8/1), stalk up to 15 × 3.5–4 μm, sterigmata gradually tapering, up to 16 × 3–3.5 μm. Basidiospores smooth, thin-walled, broadly cylindrical to narrowly ovoid, slightly to moderately curved, (7.4–) 7.8–11.0 (–11.1) × (4.3–) 4.6–5.8 (–6.2) μm (n = 30/1), L = 9.10, W = 5.21, Q’ = (1.4–) 1.5–1.9 (–2.0), Q = 1.75.

#### Remarks.

The species was described as a member of *Ductifera* (Lowy 1982), although effused basidiocarps and pedunculate basidia of *D.elastica* indicate that *Bourdotia* could have been more appropriate for it. Roberts (2003) studied the type of *D.elastica* and concluded that it is conspecific with the European Exidiopsis (Bourdotia) galzinii (see under *Protohydnumgalzinii* below). We rechecked the aforementioned type and concluded that *D.elastica* is similar to *B.galzinii* but cannot be considered its synonym. The main difference between these species is the spore width and shape – the basidiospores of *D.elastica* are certainly narrower and more regularly cylindrical than in *B.galzinii*. We therefore combine *D.elastica* in *Protohydnum* as a species of its own. The species is so far known only from the type locality in the Brazilian Amazon, and no verified sequences of *P.elasticum* currently exist.

### ﻿ Protohydnum elevatum

Taxon classificationFungiAuriculariales

V. Malysheva & Spirin sp. nov.

26668B3C-8B93-5EEE-B5BD-6733C0350D26

858668

Fig. 11D

#### Holotype.

Russia. Sakhalin Reg.: Kunashir, *Actinidiakolomikta* (dry branch), 17.VIII.2017 Bulakh (LE F-347688*, isotype – H).

#### Etymology.

Elevatus (Lat., adj.) – elevated; referred to the basidiocarp margin.

#### Description.

Basidiocarps adpressed-orbicular to cerebriform, erumpent, up to 0.5 cm in diam., first solitary, then partly fusing together, gelatinous, semitranslucent, ivory-yellowish or pale ochraceous, up to 1 mm thick, in dry condition brownish and crustaceous, margin elevated, adnate; lobes poorly pronounced, rounded, entire, 0.5–1 mm thick. Hyphal structure monomitic, hyphae hyaline, clamped, thin-walled, easily collapsing; context hyphae interwoven, 2.5–5 μm in diam., embedded in gelatinous matrix, subhymenial hyphae ascending or interwoven, rather loosely arranged, 2–4 μm in diam. Gloeocystidia abundant, hyaline to yellowish, often distinctly tapering to the apex, embedded, (23–) 26–46 (–51) × (5.2–) 5.3–7.8 (–8.1) μm (n = 20/1). Hyphidia abundant, richly branched, 1–1.5 μm in diam. at the apex, forming a continuous, loose layer up to 15 μm thick. Basidia four-celled, longitudinally septate, ovoid-ellipsoid, sessile, often distinctly tapering to the base, embedded, (18–) 24–34 (–40) × (11.8–) 12.0–15.9 (–16.7) μm (n = 20/1), sterigmata gradually tapering, up to 25 × 4–6 μm. Basidiospores smooth, thin-walled, ellipsoid-ovoid to broadly cylindrical, more rarely cylindrical and slightly curved, (11.0–) 11.8–15.7 (–16.2) × (7.4–) 7.8–10.1 (–11.1) μm (n = 30/1), L = 13.16, W = 8.91, Q’ = (1.2–) 1.3–1.7 (–1.8), Q = 1.49.

#### Distribution and ecology.

East Asia (Russia – Kuril Ids.); dead angiosperm wood.

#### Remarks.

*Protohydnumelevatum* is a close relative of *P.lactescens* from North America (Figs 1, 2). It differs from the latter species in having larger basidia and wider basidiospores, as well as shorter gloeocystidia. The species is currently known only from the type locality in East Asia.

### ﻿ Protohydnum erumpens

Taxon classificationFungiAuriculariales

A. Savchenko & Spirin sp. nov.

EDF0EC73-9779-5ACD-82FF-2BD18CE59D38

858669

Figs 6E , 11E

#### Holotype.

Kenya. Taita-Taveta: Taita Hills, Ngangao Forest, *Xymalosmonospora* (fallen branch), 23.XI.2017 Savchenko 171123/1515* (H7008774).

#### Etymology.

Erumpens (Lat., present participle of ‘erumpo’) – erumpent, in reference to the basidiocarp’s growth.

#### Description.

Basidiocarps effused, up to 3 cm in widest dimension, erumpent, smooth, gelatinous, semitranslucent, bluish-greyish to pale ochraceous, 0.04–0.1 mm thick, in dry condition almost invisible, margin gradually thinning-out. Hyphal structure monomitic, hyphae hyaline or yellowish, clamped; subicular hyphae slightly thick-walled, subparallel, frequently anastomosing, 2–3 μm in diam., subhymenial hyphae thin- to slightly thick-walled, interwoven or ascending, rather loosely arranged, 2–4 μm in diam. Cystidia absent. Hyphidia abundant, richly branched, 0.5–1 μm in diam. at the apex, occasionally forming a continuous layer up to 15 μm thick. Basidia four-celled, longitudinally septate, ovoid-ellipsoid, pedunculate, (12–) 13–16.5 (–17) × (9.0–) 9.2–11.2 (–11.3) μm (n = 20/1), stalk up to 14 × 3–4.5 μm, sometimes strongly reduced, sterigmata gradually tapering, up to 20 × 2–3 μm. Basidiospores smooth, thin-walled, cylindrical to broadly cylindrical, slightly to moderately curved, (8.9–) 9.3–12.3 (–13.4) × (5.0–) 5.1–6.4 (–6.8) μm (n = 30/1), L = 11.12, W = 5.78, Q’ = (1.6–) 1.7–2.2 (–2.3), Q = 1.93.

#### Distribution and ecology.

Africa (Kenya); partly corticated fallen angiosperm branch.

#### Remarks.

Morphologically and phylogenetically, *P.erumpens* is closest to *P.livescens* (Fig. 1). It differs from this species in having thinner basidiocarps, shorter basidia, and slightly smaller basidiospores. *Protohydnumerumpens* is so far known only from the type locality in Africa, where it was collected from angiosperm wood remnants. In turn, the European species *P.livescens* seems to be restricted to coniferous hosts.

### ﻿ Protohydnum galzinii

Taxon classificationFungiAuriculariales

(Bres.) Spirin & R.H. Nilsson comb. nov.

A07DABD0-29E7-51A8-A81F-3B5F219098D6

858679

Figs 7D , 11F

≡ Sebacinagalzinii Bres., Annales Mycologici 6: 46, 1908. Lectotype (selected by Wells and Raitviir 1975: 908). France. Aveyron: Saint-Sernin-sur-Rance, Fraxinusexcelsior, V.1905 Galzin 3832 (S F19700, studied).
= Bourdotiagalzinii (Bres.) Torrend, Brotéria Serie Botanica 11: 88, 1913.
= Bourdotiacaesia Bres. & Torrend, Brotéria Serie Botanica 11: 88, 1913. Lectotype (selected here, MBT10025860). Portugal. Lisboa, ‘ad ligna’, 27.XII.1909 Torrend (PC0084209, studied).

#### Description.

Basidiocarps effused, covering a few cm, smooth or indistinctly tuberculate, gelatinous, first semitranslucent, bluish-greyish, then opalescent, whitish, 0.1–1 mm thick, in dry condition vinaceous-brown to brownish-black, vernicose, margin gradually thinning-out. Hyphal structure monomitic, hyphae hyaline or yellowish, clamped; subicular hyphae thin-walled, subparallel, 3–4 μm in diam., subhymenial hyphae thin-walled, interwoven or ascending, rather densely arranged, 1.5–3.5 μm in diam. Gloeocystidia abundant, brownish-yellowish, deeply rooted, usually tapering to the apex, often sinuous, embedded, (68–) 70–109 (–118) × (4.1–) 4.2–7.2 (–8.6) μm (n = 30/3). Hyphidia abundant, richly branched, 1–1.5 μm in diam. at the apex, forming a continuous layer up to 20 μm thick. Basidia four-celled, longitudinally or occasionally obliquely septate, ovoid-ellipsoid, pedunculate, (12–) 13–20 (–23.5) × (8.7–) 8.9–13.1 (–14.0) μm (n = 41/4), stalk up to 16 × 2.5–3 μm, sterigmata tubular, gradually tapering, up to 20 × 2–3.5 μm. Basidiospores smooth, thin-walled, ellipsoid-ovoid to broadly cylindrical, the longest spores slightly curved, (8.1–) 8.3–12.3 (–12.8) × (5.0–) 5.1–7.3 (–7.4) μm (n = 180/6), L = 9.32–11.26, W = 6.23–6.48, Q’ = (1.2–) 1.3–2.0 (–2.3), Q = 1.49–1.75.

#### Distribution and ecology.

Europe (Austria, France, Germany, Italy, Portugal, Russia, Spain, and Ukraine), Macaronesia (Canary Islands), and Asia (Iran); decorticated wood of deciduous trees, rarely conifers.

#### Remarks.

This species was first described as a member of the simultaneously introduced subgenus Bourdotia Bres. in the genus *Sebacina* Tul. & C. Tul. (Bresadola 1908). Five years later, the subgenus was raised to the genus rank, and another species, *B.caesia*, was added (Torrend 1913). We studied type material of both species and concluded that they are conspecific. Here we treat *Bourdotia* as a synonym of *Protohydnum* and therefore transfer *B.galzinii* to the latter genus.

*Protohydnumgalzinii* is a temperate species widely distributed in Europe. Due to its prominent gloeocystidia and stalked basidia, it can be easily identified under the microscope. The identity of *B.galzinii* collections from North and South America, as well as that of *Bourdotiapetiolata* (D.P. Rogers) K. Wells, originally described from the Marshall Islands, remains obscure due to the lack of recent sequenced material. Bourdotiagalziniif.microcystidiata Hauerslev was described based on a single collection (*Galzin 14319*, PC) initially identified by Bourdot as *Sebacinamesomorpha* Bourdot & Galzin (Hauerslev 1993). We restudied this specimen and concluded that it belongs to *Exidiopsisopalea* (Bourdot & Galzin) D.A. Reid.

### ﻿ Protohydnum glabrum

Taxon classificationFungiAuriculariales

(Möller) Spirin comb. nov.

1F7E12E8-72A5-5499-BD2E-81DAB1C16996

858680

≡ Exidiopsisglabra Möller, Botanische Mittheilungen aus den Tropen 8: 168, 1895. Lectotype (selected here, MBT 10025861). Brazil. Santa Catarina, Blumenau, 8.II.1893 Möller 1039 (HBG, studied).

#### Description.

Basidiocarps effused, covering a few cm, smooth, gelatinised, semitranslucent, dirty ochraceous to brownish, 0.1–0.2 mm thick, margin gradually thinning-out. Hyphal structure monomitic, hyphae hyaline, clamped; subicular hyphae indiscernible, subhymenial hyphae thin-walled, ascending, easily collapsing, 2.5–3.5 μm in diam. Gloeocystidia yellowish, tapering, embedded, 41–69 × 6.5–12 μm. Hyphidia abundant, richly branched, 1–1.5 μm in diam. at the apex, forming a continuous layer up to 20 μm thick. Basidia four-celled, longitudinally septate, ovoid-ellipsoid, pedunculate, 14.5–18 × 9.1–12.8 μm (n = 6/1), stalk up to 17 × 3–3.5 μm, sometimes strongly reduced, sterigmata gradually tapering, up to 12.5 × 3 μm. Basidiospores smooth, thin-walled, broadly ellipsoid to subglobose, 8.0–10.2 × 6.7–7.9 μm (n = 3/1), widely collapsed.

#### Remarks.

This species was described by Möller (1895) as a member of *Exidiopsis* from the southern part of Brazil and never mentioned thereafter. Here we reassess it based on the morphological study of the single remaining specimen in the HBG herbarium (designated as the lectotype above). The completely effused, smooth basidiocarps, abundant gloeocystidia, and ovoid-ellipsoid, clearly stalked basidia point towards *Bourdotiagalzinii* (= *Protohydnumgalzinii* in the present study) and satellite species as closest to *P.glabrum*. Among these species, *P.nudum* from East Africa looks most similar to *P.glabrum*. These species can be separated based on the length of gloeocystidia (twice as long in *P.nudum* compared to *P.glabrum*) and the presence of distinct, thick epihymenial layer of hyphidia in *P.glabrum*. *Protohydnumglabrum* is so far known only from the type locality, and newly collected material is highly desirable to study it with DNA methods.

### ﻿ Protohydnum lactescens

Taxon classificationFungiAuriculariales

(Burt) Spirin & V. Malysheva comb. nov.

A16CEE29-384B-5E26-A911-5CA839917D31

858681

Figs 10A , 11G

≡ Sebacinalactescens Burt, Annals of the Missouri Botanical Garden 13: 336, 1926. Holotype. Grenada. Grand Etang, [angiosperm branch], 1912–1913 Thaxter 153 (FH00488322, studied).

#### Description.

Basidiocarps first adpressed-orbicular, up to 1 cm in diam., gelatinous, semitranslucent, pale ochraceous or greyish to brownish, 1–2 mm thick, then fusing together and forming compound effused basidiocarps up to 5 cm in diam., in dry condition brown and rather tough, margin adnate or partly detaching. Hyphal structure monomitic, hyphae hyaline, clamped; context hyphae thin-walled, interwoven or subparallel, anastomosing, embedded in gelatinous matrix, 2–4 μm in diam., subhymenial hyphae thin-walled, ascending or interwoven, 1.5–3 μm in diam. Gloeocystidia abundant, hyaline to yellowish or brownish, tapering or more rarely tubular-clavate, embedded or only slightly projecting (up to 10 μm) above hymenial layer, 48–112 × 5–11 μm. Hyphidia abundant, richly branched, 1–2 μm in diam. at the apex, forming a continuous layer up to 25 μm thick. Basidia four-celled, longitudinally septate, ovoid-ellipsoid, sessile or rarely with a reduced stalk up to 4 × 3 μm, (18–) 19–26.5 (–29) × (10.8–) 11.7–16.0 (–18.0) μm (n = 30/3), sterigmata gradually tapering, up to 20 × 3.5–4 μm. Basidiospores smooth, thin-walled, cylindrical to broadly cylindrical, occasionally slightly curved, (9.4–) 9.9–15.6 (–16.8) × (5.8–) 5.9–8.3 (–8.9) μm (n = 90/3), L = 13.05–14.04, W = 6.77–7.33, Q’ = (1.4–) 1.5–2.4 (–2.5), Q = 1.78–1.99.

**Figure 10.**
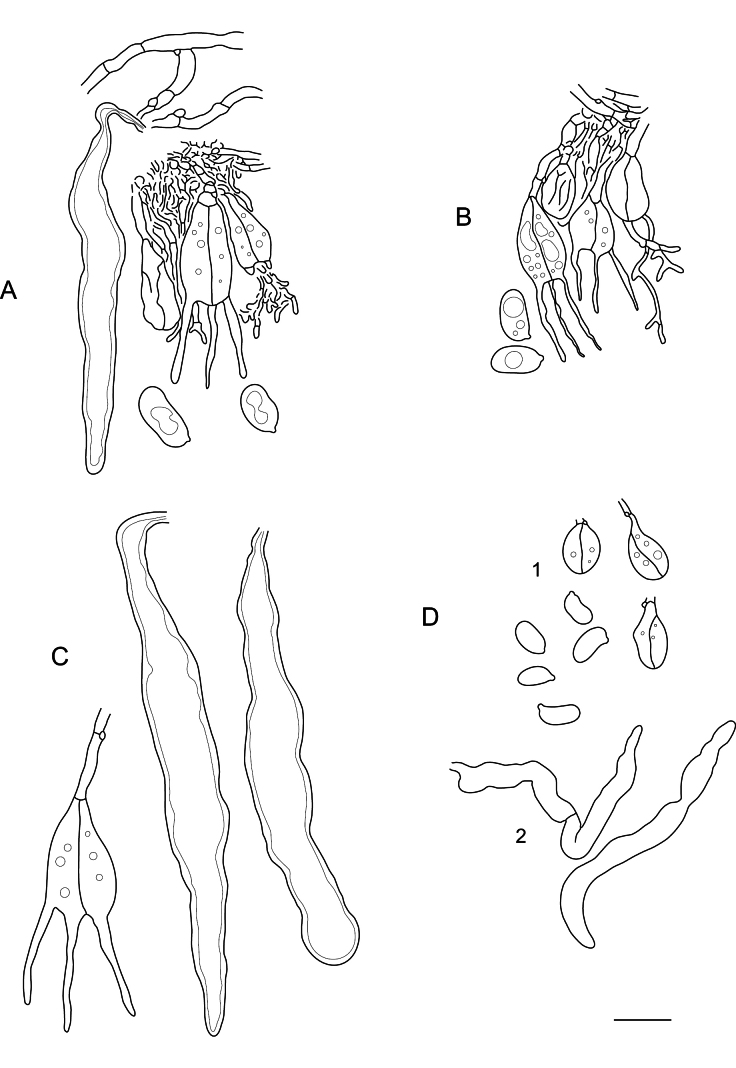
Microscopic structures of: **A.***Protohydnumlactescens* (specimen TAAM192048) (hymenial cells and tramal hyphae); **B.***P.livescens* (specimen Spirin 13913) (hymenial cells and subhymenial hyphae); **C.***P.pallidum* (holotype) (gloeocystidia and a basidium); **D.***P.sucinum* (lectotype) (1 – basidia and basidiospores; 2 – gloeocystidia). Scale bar: 10 µm.

#### Distribution and ecology.

North America (USA – California, the Caribbean – Grenada); decayed angiosperm wood.

#### Remarks.

This species was originally described from the Caribbean (Burt 1926) and later placed among the synonyms of *Ductiferasucina* (Wells 1957). We restudied the type specimens of both *Sebacinalactescens* and *Exidiasucina* (see under *Protohydnumsucinum* below) and concluded that they belong to two different species. *Protohydnumlactescens* produces effused basidiocarps, while they are cerebriform-exidioid in *P.sucinum*. Microscopically, the species can be easily separated based on size of gloeocystidia (much shorter in *P.lactescens* than in *P.sucinum*), basidia, and basidiospores (considerably larger in *P.lactescens* than in *P.sucinum*). DNA sequences of *D.sucina* published by Weiß and Oberwinkler (2001) and Wells et al. (2004) represent *P.lactescens* instead. Wells (1957) listed *Exidiacystidiata* Olive as another synonym of *D.sucina*. We did not study its type; as we could judge from the protologue, it is likely that *E.cystidiata* is a synonym of *P.lactescens*.

**Figure 11.**
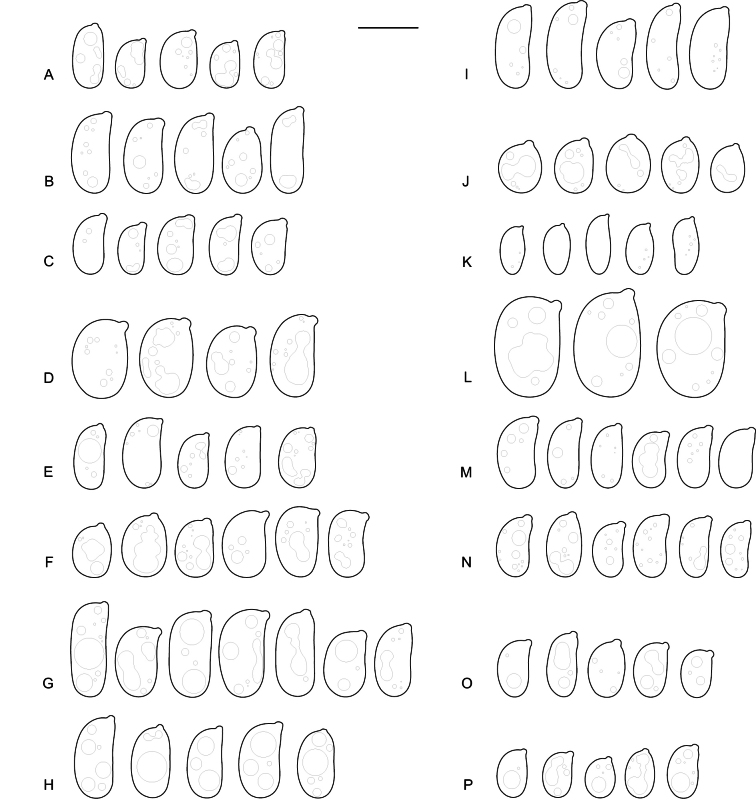
Basidiospores of: **A.***Protohydnumalbum* (specimen Miettinen 196583); **B.***P.aureum* (holotype); **C.***P.elasticum* (holotype); **D.***P.elevatum* (holotype); **E.***P.erumpens* (holotype); **F.***P.galzinii* (three left – specimen Miettinen 15900.4, three right – lectotype of *S.galzinii*); **G.***P.lactescens* (four left – specimen TAAM192048, three right – holotype of *S.lactescens*); **H.***P.livescens* (specimen Spirin 13913); **I.***P.microperum* (holotype); **J.***P.nudum* (holotype); **K.***P.ocellatum* (holotype); **L.***P.pallidum* (holotype); **M.***P.pululahuanum* (three left – lectotype, three right – specimen FH00783538); **N.***P.sucinum* (three left – lectotype of *A.brasiliensis*, three right – specimen BPI 701268); **O.***Protomeruliusamiliavi* (holotype); **P.***P.deceptorius* (holotype). Scale bar: 10 µm.

### ﻿ Protohydnum livescens

Taxon classificationFungiAuriculariales

(Bres.) Spirin & V. Malysheva comb. nov.

EFE660F7-E820-5B45-B1E1-448D44B06055

858682

Figs 6F , 10B , 11H

≡ Sebacinalivescens Bres., Fungi Tridentini 2 (11–13): 64, 1898. Lectotype (selected here, MBT 10025862). Italy. Trentino-Alto Adige: Trento, Andalo, Abiesalba (rotten log), VIII.1896 Bresadola (S F29132, studied).
= Exidiopsislivescens (Bres.) Bourdot & Maire, Bulletin de la Société Mycologique de France 36: 71, 1920.
= Sebacinalaccata Bourdot & Galzin, Bulletin de la Société Mycologique de France 39: 262, 1924. Lectotype (selected by Hauerslev 1993). France. Aveyron: Millau, L’Hospitalet-du-Larzac, Pinus sp., 25.IV.1910 Galzin 5743 (herb. Bourdot 7199) (PC, studied).

#### Description.

Basidiocarps effused, covering a few cm, smooth or indistinctly tuberculate, gelatinous, first semitranslucent, whitish-greyish or cream-coloured, then greyish- to reddish-brown, opalescent, 0.02–1 mm thick, in dry condition almost invisible or turning to a vinaceous-brown vernicose crust, margin rather sharply delimited, adnate. Hyphal structure monomitic, hyphae hyaline or yellowish, clamped; subicular hyphae thin- to slightly thick-walled, interwoven or subparallel, frequently anastomosing, 2–4 μm in diam., subhymenial hyphae thin- to slightly thick-walled, interwoven or ascending, rather densely arranged and partly glued together, 1–3.5 μm in diam. Cystidia absent. Hyphidia abundant, richly branched, 0.5–2 μm in diam. at the apex, usually forming a continuous layer up to 20 μm thick. Basidia four-celled, longitudinally or rarely obliquely septate, ovoid-ellipsoid, pedunculate, (13–) 14–20 (–22) × (9.7–) 9.8–14.3 (–14.5) μm (n = 72/7), stalk up to 15 × 2–4 μm, sometimes strongly reduced, sterigmata tubular, gradually tapering, up to 20 × 2–3.5 μm. Basidiospores smooth, thin-walled, cylindrical to broadly cylindrical, often slightly curved, (9.1–) 9.2–15.8 (–15.9) × (4.7–) 5.0–7.2 (–7.3) μm (n = 240/8), L = 11.34–13.65, W = 5.71–6.46, Q’ = (1.6–) 1.7–2.6 (–2.8), Q = 1.85–2.23.

#### Distribution and ecology.

Europe (Austria, France, Greece, Italy, Romania, Slovenia, Spain, and Ukraine); rotten decorticated wood of conifers (*Abies*, *Pinus*).

#### Remarks.

The species was originally introduced as a member of *Sebacina* accompanied by a peculiar conidial stage, *Dendrodochiumlivescens* Bres. (Bresadola 1898). The authentic material is currently stored in two parcels in Stockholm. We studied them both and concluded that these specimens belong to two different species. The *Dendrodochium* stage is certainly an asexual (*Leucogloea*) stage of a *Helicogloea* species (cf. Kirschner 2004, Spirin et al. 2018b); we will address its identity on a separate occasion. The teleomorphic fungus does represent a genuine member of the Auriculariales with four-celled, petiolate basidia, and it is evidently conspecific with *Exidiopsislaccata* Bourdot & Galzin. According to DNA data, *S.livescens* belongs to *Protohydnum*, and it is most closely related to *P.erumpens* from Africa and two neotropical species, *P.cartilagineum* (the generic type of *Protohydnum*) and *P.ocellatum* described below. The lack of gloeocystidia and longer basidiospores differentiate *P.livescens* from *P.galzinii*, another representative of the genus in central and southern parts of Europe. The lectotype of *S.laccata* described from southern France (Bourdot and Galzin 1924) is morphologically indistinguishable from the type of *S.livescens* and other specimens studied by us; *S.laccata* is therefore treated here as a synonym of *P.livescens*.

Neuhoff (1936) misapplied the name *S.livescens* to another species, which is distributed in northern Eurasia. The correct name for the latter taxon is *Exidiopsissuccinea* K. Wells & Raitviir (see description in Wells and Raitviir 1987). It differs from *P.livescens* in having sessile (not pedunculate) basidia, larger basidiospores, and it occurs on angiosperms, preferably on wood of Salicaceae. Phylogenetically, these species are not closely related (Fig. 1). All previous records of *E.livescens* from northern Europe seem to refer to *E.succinea*.

### ﻿ Protohydnum microperum

Taxon classificationFungiAuriculariales

(Kalchbr. & Cooke) Spirin comb. nov.

2111718E-CF20-5318-814A-2567A9C2901E

858683

Fig. 11I

≡ Tremellamicropera Kalchbr. & Cooke, Grevillea 9 (49): 18, 1880. Holotype. South Africa. Eastern Cape: Blue Crane Route, Somerset East, on branches, [no collecting date] MacOwan 1351 (K(M) 56709, studied).

#### Description.

Basidiocarps cushion-shaped, gregarious, erumpent, ca. 1 mm in diam., gelatinous, semitranslucent, amber-coloured, up to 0.5 mm thick, in dry condition reddish-brown. Hyphal structure monomitic, hyphae hyaline, clamped, thin- or moderately thick-walled, ascending, 1.5–2.5 μm in diam., context hyphae not differentiated. Gloeocystidia abundant, hyaline or brownish, gradually tapering to the apex, embedded or slightly projecting, 32–83 × 4.7–9.9 μm (n = 10/1). Hyphidia abundant, richly branched, 1–1.5 μm in diam. at the apex, forming a continuous layer up to 20 μm thick. Basidia four-celled, longitudinally septate, ovoid-ellipsoid, sessile, embedded, 15–23.5 × 9.8–14.2 μm (n = 12/1), sterigmata gradually tapering, up to 20 × 2.5–3 μm. Basidiospores smooth, thin-walled, cylindrical, slightly or distinctly curved, (9.8–) 10.3–15.3 (–15.8) × (4.5–) 4.6–6.5 (–6.6) μm (n = 30/1), L = 13.31, W = 5.62, Q’ = (1.8–) 1.9–2.8 (–3.3), Q = 2.39.

#### Remarks.

Anatomically, *P.microperum* shows a certain similarity to *P.aureum*. These species can primarily be separated based on basidiocarp morphology (cushion-shaped and non-fusing in *P.microperum* versus pustulate and soon coalescing in *P.aureum*) and the basidiospore width (spores in *P.microperum* are on average narrower than in *P.aureum*). *Protohydnummicroperum* is currently known only from the type locality in South Africa. Wells (1958) moved the species to *Ductifera* and treated *Seismosarcatomentosa* Olive, described from Georgia (USA), as its later synonym. As we could judge from the protologue (Olive 1947), *S.tomentosa* has a completely different basidiocarp configuration (effused, strongly darkening after drying, and possessing a white tomentose margin) and much larger basidiospores than *P.microperum*. It seems that the description of *D.micropera* by Wells (1957) refers mainly to *S.tomentosa*. After studying the type material of *P.microperum*, we found that they could hardly be conspecific; *S.tomentosa* certainly deserves a closer study.

### ﻿ Protohydnum nudum

Taxon classificationFungiAuriculariales

Spirin & Ryvarden sp. nov.

8733A9A1-7E9F-5169-AD0B-4BE93C90A0BF

858670

Fig. 11J

#### Holotype.

Kenya. Western Province: Kakamega Forest, decayed wood, 25–27.I.1973 Ryvarden 9435* (O, isotype – H).

#### Etymology.

Nudus (Lat., adj.) – nude; in reference to exposed basidia.

#### Description.

Basidiocarps effused, up to 4 cm in widest dimension, smooth, gelatinous, semitranslucent, bluish-greyish to brownish, 0.1–0.2 mm thick, in dry condition light grey and rather sturdy, opaque, margin gradually thinning-out. Hyphal structure monomitic, hyphae hyaline, clamped; subicular hyphae thin-walled or with variably thickened gelatinised walls, subparallel or interwoven, glued together, 2.5–4 μm in diam., subhymenial hyphae thin-walled, predominantly ascending, often glued, 2–4 μm in diam. Gloeocystidia abundant, hyaline to yellowish, deeply rooted or arising from subhymenial hyphae, slightly tapering to the apex, often sinuous, normally embedded, 30–154 × 3.5–6.5 μm. Hyphidia abundant, richly branched, 0.5–1 μm in diam. at the apex, normally scattered among basidia, in senescent hymenium sometimes forming a continuous layer up to 10 μm thick. Basidia four-celled, longitudinally septate, ovoid-ellipsoid, pedunculate, (17–) 17.5–23 (–24) × (10.2–) 10.3–12.4 (–12.8) μm (n = 20/2), often exposed, stalk up to 20 × 3.5–6 (–8) μm, sterigmata tubular, gradually tapering, up to 18 × 2–3.5 μm. Basidiospores smooth, thin-walled, ellipsoid-ovoid to broadly ellipsoid or more rarely subglobose, (7.2–) 7.3–10.2 (–10.6) × (5.7–) 6.2–8.9 (–9.0) μm (n = 60/2), L = 8.65–9.29, W = 6.86–7.91, Q’ = 1.1–1.4 (–1.5), Q = 1.18–1.26, apiculus occasionally eccentric.

#### Distribution and ecology.

Africa (Kenya); decorticated wood of deciduous trees.

#### Remarks.

Phylogenetically, *P.nudum* is closely related to *P.galzinii* (Fig. 2). It differs from the latter species in having wider, predominantly ellipsoid basidiospores and larger basidia. Moreover, basidial cells of *P.nudum* are often exposed, while they are normally rather deeply embedded in the layer of hyphidia in *P.galzinii*. *Protohydnumnudum* is known only from two collections from East Africa (Kenya); the range of *P.galzinii* lies further to the north, stretching from Macaronesia to the Caucasus and Middle East Asia.

### ﻿ Protohydnum ocellatum

Taxon classificationFungiAuriculariales

Alvarenga & K.H. Larss. sp. nov.

1C5E0FE1-9DC5-5834-A6F8-2994885C7EA2

858671

Fig. 11K

#### Holotype.

Brazil. Rondônia: Porto Velho, Cunião Ecological Station, angiosperm wood in lowland rain forest, 13.III.2012 Larsson 15431* (URM).

#### Etymology.

Ocellatus (Lat., adj.) – ocellate; in reference to abundant mineral inclusions.

#### Description.

Basidiocarps effused, up to 10 cm in widest dimension, tuberculate, gelatinous, semitranslucent, amber-yellow to ochraceous-reddish, 0.5–1 mm thick, containing numerous small whitish grains, in dry condition vinaceous-brown, vernicose, margin sharply delimited, adnate or partly detaching. Hyphal structure monomitic, hyphae hyaline, clamped, thin-walled, homogeneous throughout, ascending, rather tightly arranged, 3–5 μm in diam. Cystidia absent. Hyphidia abundant, richly branched, 1–1.5 μm in diam. at the apex, usually forming a continuous layer up to 15 μm thick. Basidia four-celled, longitudinally septate, ovoid-ellipsoid, pedunculate, (12–) 13–14.5 (–15) × (8.2–) 8.8–11.1 (–11.3) μm (n = 20/1), stalk up to 30 × 3–5 μm, sterigmata gradually tapering, up to 12 × 3–4 μm. Basidiospores smooth, thin-walled, cylindrical to subfusiform, (7.7–) 7.9–10.2 (–10.3) × (3.1–) 3.6–4.8 (–4.9) μm (n = 30/1), L = 8.91, W = 4.07, Q’ = (1.8–) 1.9–2.6 (–2.7), Q = 2.20, cytoplasm usually aguttulate.

#### Distribution and ecology.

South America (Brazil); decayed angiosperm wood.

#### Remarks.

*Protohydnumocellatum* is introduced here as a sibling species of *P.cartilagineum* (Figs 1, 2). The species are confusingly similar under the microscope, and they are phylogenetically closely related. The only reliable morphological difference is the hymenophore construction: the hymenophore is nearly smooth in *P.ocellatum* but consists of robust (up to 3 mm long), regularly distributed spines in *P.cartilagineum*. Nevertheless, this striking macroscopic difference and the substantial genetic distance (2.6% difference in the LSU region) allow us to distinguish between these two species. *Protohydnumocellatum* has so far only been found in the type locality in Brazil.

### ﻿ Protohydnum pallidum

Taxon classificationFungiAuriculariales

Spirin & Ryvarden sp. nov.

4DBA027E-7AEE-5AE1-AA84-011F92ABCD07

858672

Figs 10C , 11L

#### Holotype.

Zimbabwe. Manicaland: Rhodes Inyanga Nat. Park, Nyazengu, corticated hardwood branch, 16.I.1989 Ryvarden 26180* (O, isotypes – H, LE).

#### Etymology.

Pallidus (Lat., adj.) – pale, in reference to pale-coloured basidiocarps.

#### Description.

Basidiocarps effused, erumpent, up to 6 cm in widest dimension, smooth or indistinctly folded, cartilagineous, opaque, cream-coloured to pale ochraceous, 1–1.5 mm thick, in dry condition brown, vernicose, margin sharply delimited, sometimes slightly elevated, adnate or partly detaching. Hyphal structure monomitic, hyphae hyaline, clamped, homogeneous throughout, ascending or interwoven, embedded in gelatinous matrix, 2–3 μm in diam. Gloeocystidia abundant, yellowish-brownish, tapering or tubular-clavate, embedded, 85–165 × 9.5–17 μm. Hyphidia abundant, richly branched, 1.5–2 μm in diam. at the apex, forming a continuous layer up to 15 μm thick. Basidia four-celled, longitudinally septate, ovoid-ellipsoid, pedunculate, (22–) 23–31 (–32) × (14.2–) 14.4–19.8 (–20.2) μm (n = 20/1), stalk up to 22 × 4–5.5 μm, sterigmata gradually tapering or apically swollen, up to 17 × 6–7 μm. Basidiospores smooth, thin-walled, ellipsoid to broadly ellipsoid, (14.0–) 14.7–17.1 (–17.2) × (10.1–) 10.2–13.0 (–13.1) μm (n = 30/1), L = 15.77, W = 11.58, Q’ = (1.1–) 1.2–1.5 (–1.7), Q = 1.37, cytoplasm with one or several large oil drops.

#### Distribution and ecology.

Africa (Zimbabwe); still corticated angiosperm branches.

#### Remarks.

The species is introduced here based on extensive material collected in southern Africa. Morphologically, *P.pallidum* is reminiscent of the Australian *Sebacinamegaspora* G.W. Martin, combined in *Ductifera* by Wells (1957). However, *Ductiferamegaspora* (G.W. Martin) K. Wells has sessile, subglobose basidia and much larger basidiospores than those of *P.pallidum* (see descriptions in Martin 1936 and Wells 1957). The identity of *D.megaspora* remains obscure.

### ﻿ Protohydnum pululahuanum

Taxon classificationFungiAuriculariales

(Pat.) Spirin comb. nov.

6EA1EFD6-FC47-577B-B908-A32C67959AA1

858684

Fig. 11M

≡ Tremellapululahuana Pat., Bulletin de la Société Mycologique de France 9: 138, 1893. Lectotype (selected here, MBT 10025863). Ecuador. Pichincha: Quito, Pululahua, decayed branches, II.1892 Lagerheim (FH00783541, studied).
= Ductiferapululahuana (Pat.) Donk, Taxon 7: 164, 1958.
= Ductiferamillei Lloyd, Mycological Writings 5: 711, 1917 (fide Wells 1957).

#### Description.

Basidiocarps first pustulate, gregarious, ca. 1 mm in diam., then adpressed-orbicular, up to 1 cm in diam., finally fusing together and forming compound crust-like, effused fructifications up to 2 cm in the widest dimension, gelatinous, semitranslucent, cream-coloured to ivory-yellowish or pale ochraceous, 1–2 mm thick, in dry condition brown and crustaceous, margin elevated, partly detaching; lobes rounded or evenly incised, hollow, 0.5–1 mm thick. Hyphal structure monomitic, hyphae hyaline, clamped, thin- to slightly thick-walled; context hyphae interwoven or subparallel, 2–6.5 μm in diam., embedded in gelatinous matrix, subhymenial hyphae predominantly ascending, rather tightly arranged, 1–3 μm in diam. Gloeocystidia abundant to rather rare, hyaline to yellowish or brownish, gradually tapering to the apex, embedded, (55–) 65–122 (–172) × (5.0–) 5.8–11.4 (–12.3) μm (n = 24/3). Hyphidia abundant, richly branched, 0.5–1.5 μm in diam. at the apex, forming a continuous layer up to 30 μm thick. Basidia four-celled, longitudinally septate, ovoid-ellipsoid, sessile, embedded, (12–) 13.5–17.5 (–18.5) × (8.9–) 9.8–13.6 (–13.8) μm (n = 24/3), sterigmata gradually tapering, up to 15 × 2–3 μm. Basidiospores smooth, thin-walled, cylindrical to broadly cylindrical, usually slightly curved, (8.4–) 8.8–11.8 (–12.3) × (4.0–) 4.2–6.5 (–6.6) μm (n = 90/3), L = 10.02–10.34, W = 5.36–5.56, Q’ = (1.5–) 1.6–2.3 (–2.8), Q = 1.85–1.93.

#### Distribution and ecology.

South America (Ecuador); thin fallen branches of angiosperms.

#### Remarks.

*Tremellapululahuana* was described from Ecuador by Patouillard and Lagerheim (1893). Four original specimens (FH) are the source of the species description above, and one of them (with the collecting date indicated on the label) is selected as a lectotype. No sequences of *T.pululahuana s. typi* are available at the moment, and we therefore combine it in *Protohydnum* based on morphological evidence, i.e. its high similarity to *P.album* and *P.aureum. Protohydnumaureum* clearly differs from both *P.album* and *P.pululahuanum* in having larger basidia and basidiospores. Basidiospores of *P.pululahuanum* are on average slightly longer and wider than in *P.album*, although their dimensions strongly overlap. Ervin (1956) pointed at macroscopic differences between *P.album* and *P.pululahuanum* (both treated under *Gloeotromera*), and her observations correspond with our evidence. Basidiocarps of *P.pululahuanum* are substantially smaller than in *P.album* and, when fully developed, produce hollow lobes. It seems that yellowish-ochraceous colouration is characteristic of *P.pululahuanum* from the very beginning (see protologue). Fructifications of *P.album* are initially white and either remain so or get yellow-brown tints at the very end of their development or after drying. Moreover, mature basidiocarps of *P.album* are cerebriform, or foliaceous, not adpressed-orbicular or effused as in *P.pululahuanum*, and they bear entire lobes.

### ﻿ Protohydnum sucinum

Taxon classificationFungiAuriculariales

(Möller) Spirin & Alvarenga comb. nov.

9C0FD7C0-18CC-5315-8C2F-D9F7473284FC

858685

Figs 10D , 11N

≡ Exidiasucina Möller, Botanische Mittheilungen aus den Tropen 8: 169, 1895. Lectotype (selected here, MBT 10025864). Brazil. Santa Catarina, Blumenau, [1892] Möller 8 (HBG, studied).
= Auriculariabrasiliensis Lloyd, Mycological Writings 5: 785, 1918. Lectotype (selected here, MBT 10025884). Brazil. [no locality and collecting date], Rick (Lloyd’s herbarium #33208) (BPI701266, studied).

#### Description.

Basidiocarps pulvinate or cerebriform, up to 1 cm in diam., occasionally fusing together and producing compound fructifications up to 4 cm in widest dimension, gelatinous, semitranslucent, pale ochraceous or amber-coloured to brownish-orange, 1.5–8 mm thick, in dry condition brown and tough, margin elevated, partly detaching; lobes rounded, blunt, entire, up to 1.5 mm thick. Hyphal structure monomitic, hyphae hyaline or yellowish, clamped, thin-walled or with a distinct wall; context hyphae predominantly interwoven, 1–3 μm in diam., occasionally inflated up to 6 μm in diam., embedded in gelatinous matrix, subhymenial hyphae ascending, rather loosely arranged, 1–3 μm in diam. Gloeocystidia abundant, brownish, distinctly tapering at the apex, sometimes subulate, embedded or variably projecting, (75–) 80–248 (–272) × (4.0–) 4.2–10.6 (–11.1) μm (n = 30/4). Hyphidia hyaline to brownish, richly branched, 0.5–1 μm in diam. at the apex, forming a continuous layer up to 50 μm thick. Basidia four-celled, longitudinally or obliquely septate, ovoid-ellipsoid, sessile or rarely with a strongly reduced stalk up to 4 × 3 μm, (11.5–) 12–16.5 (–17) × (7.8–) 8.1–12.4 (–13.7) μm (n = 40/4), sterigmata gradually tapering, up to 30 × 3–4 μm. Basidiospores smooth, thin-walled, cylindrical to broadly cylindrical, usually slightly curved (bean-shaped), (8.3–) 8.8–11.0 (–11.1) × (4.7–) 4.8–6.1 (–6.3) μm (n = 120/4), L = 9.99–10.75, W = 5.23–5.53, Q’ = (1.5–) 1.6–2.4 (–2.6), Q = 1.83–1.99.

#### Distribution and ecology.

South America (southern part of Brazil); decayed wood of angiosperms.

#### Remarks.

The authentic specimen of *E.sucina*, as well as many other of Möller’s collections, was noted as being present in the HBG herbarium by Friedrichsen (1977) but never properly investigated. We studied it and formally designated the specimen as a lectotype of *E.sucina*. Morphologically, *P.sucinum* is most similar to *P.lactescens*; their differences are listed under that species. Three original specimens of *Auriculariabrasiliensis* (all collected by Rick in Brazil) in Lloyd’s herbarium (BPI) belong to *P.sucinum sensu typi*; we therefore place *A.brasiliensis* among the synonyms of *P.sucinum*. No modern specimens of *P.sucinum* are known to us.

### ﻿ Protohydnum

Taxon classificationFungiAuriculariales

sp. Vlasák 1808/145

3CDF2B89-81F3-5B53-83C8-6B09EF69663E

#### Description.

Basidiocarps effused, up to 4 cm in widest dimension, smooth, cartilagineous, opaque, cream-coloured, with pinkish or brownish stains, 0.5–1 mm thick, in dry condition greyish, often with ochraceous or brownish spots, crust-like, margin sharply delimited, adnate or partly detaching. Hyphal structure monomitic, hyphae hyaline, clamped, thin- or slightly thick-walled, homogeneous throughout, tightly interwoven, and strongly glued together, 1.5–2.5 μm in diam. Cystidia absent. Probasidia clavate, embedded, 12–15 × 6–7 μm. Mature basidia and basidiospores not observed.

#### Remarks.

In our phylogenetic analyses, this unnamed taxon appears as a sister species of *P.cartilagineum* and *P.ocellatum* (Figs 1, 2). However, the single studied specimen collected in French Guiana is sterile, and we therefore leave it without a formal description.

### ﻿ Protomerulius

Taxon classificationFungiAuriculariales

Möller, Botanische Mittheilungen aus den Tropen 8: 129, 1895.

BF1E4600-E6EF-5302-A0DC-01278C8F497C

#### Note.

For a treatment of this genus, see Spirin et al. (2019c).

#### Type species.

*Protomeruliusbrasiliensis* Möller.

This genus was recently amended to include half-pileate poroid, effused odontioid, and corticioid taxa possessing stalked basidia and firm hyphae; these hyphae enter into a hymenial layer as thick-walled cystidia in effused species or form an axis of tube dissepiments in poroid species (Spirin et al. 2019c). Here we describe two more corticioid species from Europe and reassess *Protomeruliuscommotus* Spirin & V. Malysheva based on more extensive material.

### ﻿ Protomerulius amiliavi

Taxon classificationFungiAuriculariales

Spirin sp. nov.

A2CB0041-CBD9-50CD-BFE4-14E9B5587EDF

858673

Figs 6G , 11O

#### Holotype.

France. Aveyron: Millau, Le Causse Noir, *Quercuspubescens* (fallen decorticated branch), 13.XI.2022 Spirin 16165* (H, isotype – PC).

#### Etymology.

From Amiliavum, the Roman name of Millau (the type locality).

#### Description.

Basidiocarps effused, smooth, waxy, greyish to greyish-ochraceous, continuous, in older parts light-brown and partly gelatinised, up to 5 cm in widest dimension, 0.1–0.2 mm thick, margin narrow, first whitish to cream-coloured, floccose, then more compact and more or less concolourous with the hymenial surface. Hyphal structure monomitic; hyphae clamped, subicular hyphae with a distinct wall to slightly thick-walled, interwoven to subparallel, 1–3 μm in diam., subhymenial hyphae thin-walled, interwoven or ascending, 1–2 μm in diam., partly glued together. Tramal cystidia abundant, hyaline or brownish, tubular-clavate, sturdy, arising from slightly thick-walled, narrow hyphae, with moderately thickened (up to 1.5 μm) walls gradually thinning-out towards the apical part, longest cystidia slightly tapering to or widened at the apex (thin-walled apical parts sometimes collapsing), (32–) 35–103 (–119) × (2.8–) 3.3–5.2 (–5.3) μm (n = 42/2), often in groups of 5–15. Hyphidia present, simple or sparsely branched, 1–1.5 μm in diam. at the apex. Crystals present, acicular or in stellate agglomerations, up to 15 μm in widest dimension. Basidia four-celled, longitudinally septate, ovoid-ellipsoid to subglobose, pedunculate, (7.8–) 8.2–11.2 (–11.8) × (6.4–) 7.1–8.8 (–9.0) μm (n = 40/2), partly glued together, stalk distinct, up to 5 × 2.5 μm, sterigmata up to 10 × 2 μm. Basidiospores ellipsoid to broadly ellipsoid, more rarely broadly cylindrical, the longest spores somewhat sigmoid, (5.9–) 6.0–9.1 (–9.4) × (3.5–) 3.7–5.1 (–5.2) μm (n = 62/2), L = 7.30–7.59, W = 4.25–4.39, Q’ = (1.3–) 1.4–2.1 (–2.3), Q = 1.67–1.80.

#### Distribution and ecology.

Europe (France); decayed angiosperm wood (*Quercus*).

#### Remarks.

Here we introduce *P.amiliavi* as a close relative of *P.brachysporus* (Bourdot & Galzin) Spirin & Malysheva. Both species possess rather thick, normally smooth basidiocarps with a gelatinised, light-brown when mature hymenium and rather narrow, fasciculate cystidia. *Protomeruliusamiliavi* differs from *P.brachysporus* in having shorter and narrower cystidia and in lacking skeletal hyphae (although they are present only in well-developed specimens of the latter species). Moreover, *P.amiliavi* was collected from rotten oak wood, and all verified records of *P.brachysporus* were collected on conifer wood.

There are two other angiosperm-dwelling European *Protomerulius* species that produce opaque and relatively thick, crust-like basidiocarps and thereby can be mistaken for *P.amiliavi*. Of them, *Protomeruliusdubius* (Bourdot & Galzin) Spirin & Malysheva is most similar to *P.amiliavi*, both macroscopically, due to its partly gelatinised and brownish basidiocarps in maturity, and microscopically, having similar cystidia and nearly identical basidiospores. However, cystidia of *P.dubius* are perceptibly wider than in *P.amiliavi*, reaching 9 μm in diam., and they are usually arranged in groups of up to eight. *Protomeruliuspertusus* Malysheva & Spirin has loose, nearly floccose basidiocarps occasionally acquiring gelatinised spots on the hymenial surface only at the very end of their development. The cystidia of *P.pertusus* are of nearly the same diameter as in *P.amiliavi*, but its basidiospores are clearly narrower (W = 3.49–4.01).

### ﻿ Protomerulius commotus

Taxon classificationFungiAuriculariales

Spirin & V. Malysheva, Mycological Progress 18: 1087, 2019.

C547F742-F3DB-5D61-8131-A8C61CCC0C13

#### Holotype.

Norway. Vestfold: Larvik, Kvelde, Jordstøyp, *Ulmusglabra* (rotten log), 15.IX.2016 Spirin 11097* (O, studied).

#### Description.

Basidiocarps effused, smooth, first pruinose-reticulate, waxy, semitranslucent, greyish, then continuous, gelatinised, greyish or brownish when old or dry, up to 2 cm in widest dimension, 0.05–0.1 mm thick, margin concolourous with hymenium, gradually thinning-out. Hyphal structure monomitic; hyphae clamped, subicular hyphae with a distinct wall, subparallel and densely packed, 1–2.5 μm in diam., subhymenial hyphae thin-walled, interwoven or ascending, 1.5–3 μm in diam., often short-celled, glued together. Tramal cystidia abundant, hyaline or brownish, tubular-clavate, sturdy, arising from thin-walled hyphae, with thickened (up to 3.5 μm) walls gradually thinning-out towards the apical part, the longest cystidia slightly tapering to or widened at the apex (thin-walled apical parts often collapsing), (54–) 57–149 (–154) × (4.2–) 4.6–9.4 (–11.3) μm (n = 101/5), single or more often in groups of 2–8, sometimes biradicate, some cystidia tortuous; hymenial cystidia hyaline, broadly clavate to subglobose, thin- or slightly thick-walled, 10–50 × 4–13.5 μm, scattered among basidia, more rarely associated with tramal cystidia. Hyphidia present, simple or sparsely branched, 1–1.5 μm in diam. at the apex. Crystals occasionally present on hyphidia and cystidia, acicular or fused in stellate agglomerations. Basidia four-celled, longitudinally septate, ovoid-ellipsoid to subglobose, pedunculate, (6.8–) 7.1–9.1 (–9.4) × (5.9–) 6.2–7.8 (–8.2) μm (n = 30/3), spaced or partly glued together, stalk distinct, up to 6 × 2.5 μm, sterigmata up to 8 × 2–2.5 μm. Basidiospores smooth, thin-walled, ellipsoid to broadly cylindrical, more rarely lacrymoid, (4.1–) 4.4–6.8 (–7.1) × (3.0–) 3.2–4.3 (–4.4) μm (n = 180/6), L = 5.24–5.95, W = 3.45–3.85, Q’ = (1.2–) 1.3–1.7 (–1.9), Q = 1.47–1.61.

#### Distribution and ecology.

Europe (France, Italy, Norway, Sweden, and Switzerland – basidiocarps on wood; Czech Republic, Estonia, Germany, and United Kingdom – soil sequences); strongly decayed wood of deciduous trees (*Carpinus*, *Fraxinus*, *Quercus*, and *Ulmus*).

#### Remarks.

*Protomeruliuscommotus* was described based on two collections from Norway as a close relative of the widely distributed *Protomeruliusmadidus* Spirin & K.H. Larss. (Spirin et al. 2019c). Here we reassess it after investigating newly collected material and detecting one more closely related species, *P.deceptorius* (described below). Macroscopically, *P.commotus* differs from the two aforementioned species due to the lack of white mineral inclusions usually detectable without a lens; however, they are present only in mature basidiocarps of those species. Both *Protomeruliuscommotus* and *P.deceptorius* have broadly clavate or bubble-like hymenial cystidia, in addition to tubular thick-walled cystidia of tramal origin. *Protomeruliusmadidus* is devoid of hymenial cystidia, and its basidiospores are on average larger than in *P.commotus* and *P.deceptorius* (see description in Spirin et al. 2019c). Differences of *P.commotus* from *P.deceptorius* are given under the latter species. *Protomeruliuscommotus* seems to be widely distributed in temperate forests of Europe (Fig. 4), but it is most likely overlooked due to its diminutive basidiocarps.

### ﻿ Protomerulius deceptorius

Taxon classificationFungiAuriculariales

Spirin & Viner sp. nov.

1A9105D9-F5B1-5813-98AD-77AFD713199A

858674

Figs 6H , 11P

#### Holotype.

Slovenia. Kočevje: Podstenice, Rajhenavski Rog, *Fagussylvatica* (rotten decorticated log), 19.VIII.2021 Spirin 14811* (H, isotype – LJF).

#### Etymology.

Deceptorius (Lat., adj.) – deceptive, in reference to small morphological differences from the closely related species.

#### Description.

Basidiocarps effused, smooth, first pruinose-reticulate, waxy, semitranslucent, greyish, then continuous, gelatinised, up to 1 cm in widest dimension, 0.03–0.07 mm thick, margin concolourous with hymenium, gradually thinning-out; tiny white spots often present in mature basidiocarps, irregularly distributed on hymenial surface. Hyphal structure monomitic; hyphae clamped, subicular hyphae with a distinct wall, subparallel, 2–3 μm in diam., subhymenial hyphae thin-walled or with a distinct wall, interwoven or ascending, 1.5–2.5 μm in diam., glued together. Tramal cystidia abundant, hyaline or brownish, tubular-clavate, usually tapering but sometimes slightly widened at the apex, arising from thin- or moderately thick-walled hyphae, with thickened (up to 3 μm) walls gradually thinning-out towards the apical part (thin-walled apical parts often collapsing), (45–) 46–124 (–127) × (5.2–) 5.3–11.3 (–11.8) μm (n = 134/7), single or in groups of 2–5, occasionally biradicate, sometimes tortuous; hymenial cystidia hyaline, broadly clavate to subglobose, thin-walled, quickly collapsing, 12–26 × 5.2–12.4 μm, scattered among basidia. Hyphidia present, simple or sparsely branched, 1–1.5 μm in diam. at the apex. Crystals occasionally present on hyphidia and cystidia, acicular or fused in stellate agglomerations. Basidia four-celled, longitudinally septate, ovoid-ellipsoid, pedunculate, (6.9–) 7.0–9.1 (–9.8) × (6.0–) 6.1–7.2 (–7.8) μm (n = 21/2), tightly glued together, stalk distinct, up to 4 × 3 μm, sterigmata up to 5 × 1.5–2 μm. Basidiospores smooth, thin-walled, ellipsoid to broadly cylindrical or cylindrical, more rarely lacrymoid, (4.6–) 4.8–6.8 (–7.2) × (3.0–) 3.1–4.7 (–4.9) μm (n = 210/7), L = 5.54–5.94, W = 3.59–4.11, Q’ = (1.2–) 1.3–1.8 (–1.9), Q = 1.44–1.63.

Ecology and distribution. Europe (Slovenia – basidiocarps on wood; Estonia, Georgia, Portugal, and Romania – soil sequences), North America (USA, West Virginia – soil sequence) (see further remarks below); strongly rotten wood of deciduous trees (*Fagus*, *Salix*).

#### Remarks.

*Protomeruliusdeceptorius* is introduced here as a close relative of *P.commotus* and *P.madidus* (Figs 1, 4). Morphologically, it is highly similar to *P.commotus* and differs from it primarily in having shorter tramal and smaller hymenial cystidia. Moreover, basidiospores of *P.deceptorius* are slightly wider than in *P.commotus*, although this difference is merely statistical, and the significant part of *P.deceptorius* specimens have basidiospores of the same size as in *P.commotus*. Mature basidiocarps of *P.deceptorius* usually contain white mineral inclusions (Fig. 6); they are absent in all *P.commotus* specimens studied by us.

In total, eleven specimens of *P.deceptorius* are known to us, all collected in Slovenia and all but one derived from rotten wood of *Fagussylvatica*. However, DNA sequences obtained from forest soil and root tips indicate that the species seems to be widely distributed in Europe (Fig. 4). GenBank sequence MF665126 from West Virginia, USA, shows a 1 bp difference in the ITS2 region *versus P.deceptorius* from Europe. We consider it as an intraspecific variation and assign this sequence to *P.deceptorius*. It was obtained from root tips of trees in a montane forest dominated by *Fagusgrandifolia* and *Quercus* spp. (Nelson 2017). Several other soil sequences from GenBank and UNITE cluster with *P.deceptorius*, although without statistical support (Fig. 4). These all originated from subtropical and tropical areas (South America, Southeast Asia, and Oceania) and may well represent sister taxa of *P.deceptorius*. On the other hand, their differences *versus P.deceptorius* sequences from Europe and North America may reflect genetic variation within one widely distributed species. Some other *Protomerulius* species with a wide distribution range (e.g., *P.brachysporus*, *P.minor*, and *P.subreflexus*) demonstrate significant ITS variation within one species (Spirin et al. 2019c). Whatever the case may be, a definite conclusion can be reached only after sequencing the full-length ITS region and, desirably, additional genetic markers from physical *P.deceptorius* s. lato specimens collected in subtropical and tropical areas.

## ﻿Discussion

In the present paper, we describe sixteen new species of Auriculariales with stalked basidia. Additionally, the generic affiliation of eleven extant species was reassessed based on phylogenetic and/or morphological data. Of these newly described or reassessed taxa, only one, *Elmericiumalabastrinum*, belongs to the core group of Auriculariales, *i.e.* the family Auriculariaceae, where it clusters with other genera that have peculiar petiolate basidia (*Elmerina* and *Protodaedalea*). The remaining genera dealt with above do not show particularly close affinity to the Auriculariaceae or to each other. We therefore refrain from proposing any novel suprageneric reclassification of these genera, pending denser taxon sampling and sequencing of additional genetic markers. In the case of the *Protodontia*–*Gelacantha* clade, new data will certainly facilitate a better understanding of generic limits in this group than we present here.

In our earlier revision of the Auriculariales with sphaeropedunculate basidia, five monotypic genera were introduced to accommodate taxa morphologically highly similar to *Myxarium* but phylogenetically isolated from it (Spirin et al. 2019b, c). In this paper, we add new species to the previously monotypic *Hydrophana*, *Mycostilla*, and *Protoacia*. However, the discovery of these so far unknown species in these recently established genera has posed another problem. Namely, expanding these genera has blurred their distinctions from other morphologically similar but phylogenetically distant genera, which were originally emphasised as unique. For example, the monotypic genus *Mycostilla* was originally distinguished from all other genera of the myxarioid Auriculariales (except *Stypellopsis* Spirin & Malysheva) by its giant, thin-walled cystidia. The introduction of another *Mycostilla* species, *M.chromatica*, which lacks cystidia entirely, makes it impossible to maintain the original concept of the genus.

We show that the presence of sphaeropedunculate basidia alone is no longer sufficient to determine the generic affiliation of a given species. Spirin et al. (2019b) demonstrated that *Microsebacinafugacissima* (Bourdot & Galzin) P. Roberts, a species with strictly sessile basidia, belongs to the genus *Myxarium*, which is otherwise represented by taxa with stalked basidia. The genus *Protohydnum*, as amended above, includes approximately the same number of species with stalked and sessile basidia. Further exploration of the diversity of the myxarioid Auriculariales will likely render the situation even more complicated. Nevertheless, we still believe that, in most cases, morphological definition of a genus is possible if based on a combination of morphological traits (cf. Spirin et al. 2024b). However, re-evaluation of taxonomically significant traits should be grounded in a densely sampled, multigene phylogeny of the corresponding suprageneric unit, *i.e.* a family or an order.

Soil and environmental sequences provide another important source of information about species diversity and distribution within the group. At least one more *Protoacia* species related to *P.delicata* and one more *Myxarium* species (potentially conspecific with *M.inconspicuum*, treated above) have been detected among unnamed GenBank sequences. Although basidiocarps of the newly described *Protomeruliusdeceptorius* have been collected only in Slovenia, available soil sequences suggest a much wider distribution for this species in Europe and North America. Additionally, at least two more undescribed *Protomerulius* species are present among environmental data: one is a close relative of *P.madidus*, distributed in North America, and another is a sister taxon of *P.minor* from Argentina (Fig. 4).

Recently, a few *Protomerulius* species have been shown to form symbiotic associations with several genera of orchids (McCormick et al. 2021; Suetsugu and Matsubayashi 2021). Thus, new data from mycorrhizal studies will likely expand our current knowledge of species diversity in *Protomerulius* spp. across different geographic regions, as well as their ecological roles and interactions with other organisms.

### ﻿Specimens examined (type specimens cited above are not listed)

Bourdotiagalziniif.microcystidiata Hauerslev. France. Aveyron: Mélagues, Le Rec, *Castaneasativa*, 13.XI.1913 Galzin 14319 (herb. Bourdot 13956) (PC, holotype).

*Elmericiumalabastrinum* Spirin & V. Malysheva. Russia. Khabarovsk Reg.: Khabarovsk Dist., Malyi Niran, *Acermono*, 7.VIII.2012 Spirin 5002 (H); Solnechnyi Dist., Suluk-Makit, *Populusmaximowiczii*, 20.VIII.2011 Spirin 4226, 4227 (H).

*Endoperplexadartmorica* P. Roberts. Estonia. Viljandimaa: Tipu, Maasaare, *Pinussylvestris*, 16.IX.2018 Spirin 12344* (H, TUF114815). Norway. Vest-Agder: Mandal, Uføra, *Piceaabies*, 2.XI.2017 Spirin 11781*, 11783* (O, H). Sør-Trøndelag: Meldal, Urvatn, *P.abies*, 27.IX.1991 Bendiksen & Høiland 9-44 (O F149952). Sweden. Västergötland: Töllsjö, rotten wood, 1.X.1969 Hjortstam 2784 (GB-0159286).

*Exidiopsisgloeophora* (Oberw.) Wojewoda. Norway. Nord-Trøndelag: Snåsa, Blåfjella, *P.abies*, 28.IX.2011 J. Nordén 9609* (O).

*Exidiopsissuccinea* K. Wells & Raitviir. Canada. British Columbia: Mc Leod Dist., road 44, *Populus* sp., 25.VI.1969 Eriksson 12078 (GB-0185809). Russia. Khabarovsk Reg.: Verkhnebureinsky Dist., Sidorka, *Salixschwerinii*, 24.VIII.2014 Spirin 7958* (H). Krasnoyarsk Reg.: Yenisseysk Dist., Yartsevo, *Salixfragilis*, 10.VIII.1958 Parmasto (TAAM 7008, holotype). Leningrad Reg.: Boksitogorsk Dist., Radogosch’, *Salixcinerea*, 26.IX.2012 Spirin 5836* (H). Sweden. Västergötland: Medelplana, Munkängarna, *Ulmusglabra*, 19.XII.2024 Spirin 18015 (GB). Torne Lappmark: Abisko Nat. Park, *Salix* sp., 8.VIII.1981 Hallenberg 3734 (GB-0185320).

*Hydrophanasphaerospora* (Bourdot & Galzin) Malysheva & Spirin. Sweden. Dalarna: Malung, Lybergsgnupen, *Betula* sp. (?), 23.VIII.2024 Sivula (GB). Halland: Särö, *Quercusrobur*, 12.X.2024 Spirin 17677 (GB). Västergötland: Göteborg, Änggården, *U.glabra*, 3.X.2024 Spirin 17632 (GB).

*Mycostillachromatica* Spirin & Grebenc. Slovenia. Kočevje: Podstenice, Rajhenavski Rog, *Abiesalba* (fallen log), 30.VII.2020 Spirin 14013 (H).

*Mycostillavermiformis* (Berk. & Broome) Spirin & Malysheva. France. Aveyron: Millau, Le Causse Noir, *Pinusnigra*, 8.V.2022 Spirin 15335* (H). Netherlands. Drenthe: Dwingeloo, Lheebroekerzand, Reigerplas, *Juniperuscommunis*, 1.VI.1970 de Vries 631 (GB-0185457). Romania. Braşov: Şinca, *A.alba*, 14.IX.2021 Spirin 14917, 14946, 14948 (H). Slovenia. Radovljica: Lipanca, *Larixdecidua*, 26.IX.2019 Spirin 13270 (H). Sweden. Bohuslän: Resteröd, Ulvesund, *P.abies*, 14.X.2023 Spirin 16921 (GB). United Kingdom. England: Somerset, Bathford Plantation, dead wood, 1.IV.1877 Broome 404 (K(M) 47312, holotype of *Dacrymycesvermiformis*).

*Myxariumcinnamomescens* (Raitv.) Raitv. Austria. Niederösterreich: Perchtoldsdorf, Naturpark Föhrenberge, *Tiliacordata*, 10.IX.2022 Spirin 15784 (H). France. Essonne: Soisy-sur-Seine, Forêt de Sénart, *T.cordata*, 11.XI.2021 Spirin 15272 (H). Russia. Sakhalin Reg.: Kunashir, *Betula* sp., 17.VIII.2017 Bulakh (LE F-347687*). Sweden. Bohuslän: Hönö, Ersdalsvägen, *Populustremula*, 15.VII.2024 Spirin 17307 (GB). Västergötland: Göteborg, Guldheden, *P.tremula*, 16.XII.2024 Spirin 17999 (GB); Hjälmsäter, Såten, *P.tremula*, 19.XII.2024 Spirin 18014 (GB); Partille, Tultered, *P.tremula*, 3.XI.2024 Spirin 17782, 17784 (GB); Vänersborg, Hunneberg, *T.cordata*, 15.X.2024 Spirin 17698 (GB); Vrångö, Vrångöskärgården, *P.tremula*, 15.XI.2024 Spirin 17987, 17990 (GB).

*Myxariumcrozalcii* (Bourdot & Galzin) Spirin & Malysheva. France. Aveyron: Millau, Le Causse Noir, *Quercuspubescens*, 21.II.2025 Spirin 18033 (H). Sweden. Skåne: Torekov, Hallands Väderö, *Fagussylvatica*, 25–27.X.1975 Hallenberg 1197 (GB-0185739). Småland: Värnamo, Åminne, *F.sylvatica*, 17.X.2024 Spirin 17758 (GB). Västergötland: Götene, Västerplana Storäng, *U.glabra*, 28.VIII.2024 Spirin 17510 (GB).

*Myxariumcrystallinum* D.A. Reid. Finland. Uusimaa: Helsinki, Oulunkyla, Veräjälaakso, *Acerplatanoides*, 17.X.2019 Viner 2023/26* (H). Norway. Sør-Trøndelag: Kongsvoll, Vårstigen, *Betulatortuosa*, 24.VIII.1979 Hjortstam 10447 (GB-0185788). Sweden. Småland: Värnamo, Rusarebo, *U.glabra*, 17.X.2024 Spirin 17736 (GB).

*Myxariumevanidum* Spirin & K.H. Larss. Finland. Varsinais-Suomi: Raasepori, Ramsholmen, *U.glabra*, 6.XI.2021 Spirin 15193* (H). Sweden. Småland: Värnamo, Åminne, *F.sylvatica*, 17.X.2024 Spirin 17761* (GB).

*Myxariumfugacissimum* (Bourdot & Galzin) Malysheva & Spirin. Finland. Uusimaa: Helsinki, Östersundom, Stora Dammen, *A.platanoides*, 31.X.2022 Spirin 16085 (H). France. Aveyron: Millau, L’Hospitalet-du-Larzac, *Q.pubescens*, 8.XI.2024 Spirin 17971* (H).

*Myxariumgrilletii* (Boud.) D.A. Reid. France. Aveyron: Millau, Creissels, *Acerpseudoplatanus*, 16.XI.2022 Spirin 16479* (H). Sweden. Småland: Högsby, Långemåla, Getebro, *F.sylvatica*, 27.IX.2024 Spirin 17598 (GB). Västergötland: Göteborg, Änggården, *U.glabra*, 7.VII.2024 Spirin 17256, 17265 (GB), 9.VII.2024 Spirin 17271 (GB); Götene, Västerplana Storäng, *Q.robur*, 28.VIII.2024 Spirin 17514 (GB), *U.glabra*, 28.VIII.2024 Spirin 17511 (GB); Hällekis, Törnsäter, *U.glabra*, 28.VIII.2024 Spirin 17520, 17526, 17528 (GB); Partille, Tultered, *P.tremula*, 3.XI.2024 Spirin 17776 (GB); Vänersborg, Västra Tunhem, *Q.robur*, 15.X.2024 Spirin 17712, 17714 (GB). Bohuslän: Hönö, Ersdalsvägen, *P.tremula*, 12.VIII.2024 Spirin 17366 (GB); Mjörn, Sundsby, *Corylusavellana*, 7.IX.2024 Spirin 17536, 17538, 17540 (GB), *F.sylvatica*, 7.IX.2024 Spirin 17531 (GB).

*Myxariumhyalinum* (Pers.) Donk. Austria. Niederösterreich: Perchtoldsdorf, Naturpark Föhrenberge, *F.sylvatica*, 10.IX.2022 Spirin 15772 (H). France. Aveyron: Millau, Creissels, *A.pseudoplatanus*, 16.XI.2022 Spirin 16482 (H). Sweden. Västergötland: Göteborg, Änggården, *U.glabra*, 7.VII.2024 Spirin 17264 (infected by *Zygogloeagemellipara* P. Roberts) (GB); Vrångö, Vrångöskärgården, *P.tremula*, 15.XI.2024 Spirin 17995 (GB). Bohuslän: Hönö, Ersdalsvägen, *Cytisusscoparius*, 12.XI.2023 Spirin 17040, 17044* (GB); Ökerö, Hälsö, *Betulapubescens*, *Hippophaerhamnoides*, 11.VII.2024 Spirin 17282, 17284 (GB).

*Myxariumlegonii* (P. Roberts) P. Roberts. France. Aveyron: Millau, Dourbie, *Fraxinusexcelsior*, 15.XI.2022 Spirin 16337 (H), Ravin de Potensac, *Populusnigra* (rotten log), 15.XI.2022 Spirin 16419* (H). Spain. Navarra: Sarasibar, rotten wood, 9.XI.2020 García (H). United Kingdom. England: Surrey, Runnymede, Cooper’s Hill, *Ulmus* sp., 30.VI.1988 Legon (TAAM 132119).

*Myxariummesomorphum* (Bourdot & Galzin) Hauerslev ex Spirin, Malysheva, P. Roberts, Trichies, A. Savchenko & K.H. Larss. France. Aveyron: Millau, Dourbie, *F.excelsior*, 23.II.2025 Spirin 18159 (H). Sweden. Västergötland: Mölndal, Solängen, Snöhöjdsliden, *Thymus* sp. (cultivated), 5.II.2024 Spirin 17047 (GB); Vänersborg, Västra Tunhem, *Q.robur*, 15.X.2024 Spirin 17717 (GB); Vrångö, Vrångöskärgården, *C.avellana*, 15.XI.2024 Spirin 17993 (GB).

*Myxariumminutissimum* (Höhn.) Spirin & Trichies. Austria. Niederösterreich: Perchtoldsdorf, Naturpark Föhrenberge, *F.sylvatica*, 10.IX.2022 Spirin 15780 (H). Denmark. Jylland: Mols Bjerge, Strandkjaer, *J.communis* (?), 25.VII.1981 Hallenberg 3501 (GB-0185500). Finland. Uusimaa: Helsinki, Vuosaari, Bröanda, *S.caprea*, 4.XI.2022 Spirin 16127 (H). Norway. Rogaland: Hogganvik, *F.sylvatica*, 2.VIII.1984 Hallenberg 8377 (GB-0185497); Ropeid, *Q.robur*, 3.VIII.1984 Hallenberg 8383 (GB-0185498). Slovenia. Postojna: Drskovče, *C.avellana*, 29.IX.2023 Spirin 16810 (H); Sežana: Divača, *Q.pubescens*, 27.IX.2023 Spirin 16683* (H). Sweden. Småland: Högsby, Långemåla, Bokhultet, *Q.robur*, 24.IX.2024 Spirin 17555 (GB), Danmarksvägen, *F.sylvatica*, 27.IX.2024 Spirin 17621 (GB), Getebro, *F.sylvatica*, 27.IX.2024 Spirin 17603, 17607, 17611 (GB); Värnamo, Rusarebo, *A.platanoides*, 17.X.2024 Spirin 17733 (GB). Västergötland: Hålanda, Holmevattnet, deciduous wood, 3.IX.1970 Hjortstam 3402 (GB-0185731); Göteborg, Änggården, *T.cordata*, 7.VII.2024 Spirin 17258 (GB), *U.glabra*, 9.VII.2024 Spirin 17270* (GB); Medelplana, Munkängarna, *U.glabra*, 15.X.2023 Spirin 16947 (GB); Partille, Tultered, *P.tremula*, 3.XI.2024 Spirin 17773 (GB). Bohuslän: Hönö, Ersdalsvägen, *C.scoparius*, 15.VII.2024 Spirin 17325 (GB); Ökerö, Hälsö, *Q.robur*, 11.VII.2024 Spirin 17303 (GB).

*Myxariumnucleatum* Wallr. Austria. Niederösterreich: Perchtoldsdorf, Naturpark Föhrenberge, *F.excelsior*, 10.IX.2022 Spirin 15781 (H). France. Aveyron: Millau, Creissels, *F.excelsior*, 16.XI.2022 Spirin 16494 (H). Sweden. Västergötland: Partille, Tultered, *P.tremula*, 3.XI.2024 Spirin 17774 (GB).

*Myxariumpodlachicum* (Bres.) Raitv. Austria. Niederösterreich: Perchtoldsdorf, Naturpark Föhrenberge, *C.avellana*, 10.IX.2022 Spirin 15795, 15796 (H), *F.sylvatica*, 10.IX.2022 Spirin 15778 (H). Finland. Varsinais-Suomi: Raasepori, Ramsholmen, *U.glabra*, 6.XI.2021 Spirin 15194 (H). Sweden. Halland: Särö, *B.pubescens*, 12.X.2024 Spirin 17670 (GB). Småland: Högsby, Långemåla, Barnebo, *U.glabra*, 23.IX.2023 Spirin 17552 (GB), Långemåla, Getebro, *F.sylvatica*, 27.IX.2024 Spirin 17609 (GB), Bokhultet, *F.sylvatica*, 24.IX.2024 Spirin 17557 (GB); Värnamo, Rusarebo, *A.platanoides*, 17.X.2024 Spirin 17722 (GB), Åminne, *F.sylvatica*, 17.X.2024 Spirin 17755 (GB). Västergötland: Göteborg, Änggården, *Alnusglutinosa*, 7.VII.2024 Spirin 17251 (GB), *U.glabra*, 7.VII.2024 Spirin 17261 (GB), 9.VII.2024 Spirin 17273 (GB); Medelplana, Munkängarna, *U.glabra*, 15.X.2023 Spirin 16934, 16945 (GB); Vänersborg, Hunneberg, *C.avellana*, 15.X.2024 Spirin 17694 (GB), Västra Tunhem, *Q.robur*, 15.X.2024 Spirin 17720 (GB); Vrångö, Vrångöskärgården, *Sorbus* sp., *C.avellana*, 19.VII.2024 Spirin 17335, 17337 (GB), Tärnstigen, *Q.robur*, 19.VII.2024 Spirin 17344, 17345 (GB). Bohuslän: Hönö, Ersdalsvägen, *P.tremula*, 15.VII.2024 Spirin 17305 (GB); Ljungskile, Bratteforsån, *U.glabra*, 17.VIII.2023 Spirin 16589* (GB); Mjörn, Sundsby, *F.sylvatica*, *Q.robur*, 7.IX.2024 Spirin 17534, 17542 (GB).

*Myxariumpopulinum* (P. Karst.) Spirin & Malysheva. Sweden. Dalarna: Malung, Bötåberget, *P.tremula*, 25.VIII.2024 Spirin 17502 (GB). Västergötland: Partille, Tultered, *P.tremula*, 3.XI.2024 Spirin 17779, 17783 (GB).

*Myxariumspiniferum* Spirin & V. Malysheva. USA. New York: Essex Co., Arbutus Lake, decayed wood, 17.VIII.2012 Miettinen 15677* (H). Tennessee: Sevier Co., Ramsey Cascades Trail, decayed wood, 13.VII.2004 Larsson 12177 (GB).

*Myxariumvarium* Hauerslev. France. Aveyron: Millau, L’Hospitalet-du-Larzac, *Q.pubescens*, 7.XI.2024 Spirin 17908 (H). Lozère: Saint-Etienne-du-Valdonnez, Forêt du Sapet, *F.sylvatica*, 28.VI.2022 Spirin 15558, 15560 (H), 29.VI.2022 Spirin 15646*, 15649 (H). Slovenia. Ilirska Bistrica: Snežnik, *F.sylvatica*, 28.IX.2023 Spirin 16770 (H). Sweden. Västergötland: Asklanda, Kvinnestad, deciduous branches, 6.X.1970 Hjortstam 5130 (GB-0185733); Vrångö, Vrångöskärgården, *P.tremula*, 19.VII.2024 Spirin 17332 (GB), Tärnstigen, *Q.robur*, 19.VII.2024 Spirin 17343 (GB). Bohuslän: Hönö, Ersdalsvägen, *C.scoparius*, 15.VII.2024 Spirin 17313 (GB); Säve, Lindesnäs, *C.avellana*, 1.XI.1973 Eriksson (GB-0185755).

*Oliveoniafibrillosa*. USA. Washington: Jefferson Co., Morgan’s Crossing, *Acermacrophyllum*, 7.X.2014 Spirin 8257* (H).

*Protoaciadelicata* Spirin & Malysheva. Finland. Inarin Lappi: Inari, Kuusipää, *P.abies*, 9.IX.1962 Strid & Eriksson 10143 (GB-0185568). Sompion Lappi: Pelkosenniemi, Iso Palovaara, *P.abies*, 5.IX.2020 Spirin 14172, 14180, 14182 (H). Norway. Nord-Trøndelag: Stjørdal, Nyvollkjølen, on wood in *Picea* forest, 15.IX.1983 Hallingbäck (GB-0185559). Russia. Nizhny Novgorod Reg.: Sharanga Dist., Kilemary, *P.abies*, 24.VIII.2019 Spirin 13010* (H). Sweden. Småland: Värnamo, Björs, *P.abies*, 26.VIII.1959 Eriksson 3925 (GB-0185749), 16.X.1960 Eriksson 3950 (GB-0185748). Västergötland: Östad, Fåglaryd, *P.sylvestris*, 4.IX.1970 Hjortstam *3491* (GB-0185732). Dalarna: Älvdalen, Rödberget, *P.abies*, 19.VIII.2024 Spirin 17383 (GB); Transrand, Bredvallen, *P.abies*, 22.VIII.2024 Spirin 17445 (GB); Garpenberg, decayed trunk, 29.VIII.1974 Hallenberg 19 (GB-0185628). Jämtland: Revsund, Stavre, *P.sylvestris*, 30.VII.1958 Eriksson 8288 (GB-0185783). Åsele Lappmark: Åsele, Rödberget, *P.abies*, 28.VIII.2023 Spirin 16601 (GB).

*Protodontiasubgelatinosa* (P. Karst.) Pilát. Russia. Nizhny Novgorod Reg.: Lukoyanov Dist., Panzelka, *T.cordata*, 1.VIII.2019 Spirin 12797 (H). Sweden. Västergötland: Göteborg, Änggården, *A.glutinosa*, 3.X.2024 Spirin 17626 (GB).

*Protohydnumalbum* (Lloyd) Spirin. USA. Arkansas: Marion Co., Buffalo Point, angiosperm, 26.X.2013 Miettinen 17498 (H). Illinois: McLean Co., Funk’s Grove, hardwood, 13.VIII.1965 Cain (LE 37320* ex TRTC). Indiana: Montgomery Co., Crawfordsville, dead logs, 5.XI.1922 Bechtel 5, 6 (BPI 719951, 719953). Iowa: Tama Co., Tama, 30.VI.1928 Shimek (BPI 719954). Minnesota: Waseca Co., Janesville, Willis Lake, *Acersaccharum*, 21.VIII.2013 Miettinen 16734.1 (H). Missouri: St. Louis Co., Allenton, *Platanus* sp. (?), 8.IX.1929 Linder (BPI 719949). Nebraska: [no locality indicated], old logs, VII.1887 Webber (BPI 719948). New York: Ontario Co., Farmington, VIII.1889 Brown (BPI 719952). Tennessee: Sevier Co., Ramsey Cascade trail, *Tiliaamericana*, 30.IX.2015 Miettinen 19583* (H).

*Protohydnumcartilagineum* Möller. Brazil. Santa Catarina: Blumenau, 1891 Möller 106 (HBG), [no collecting date] Möller 19, 20 (HBG). São Paulo: Iguape, Jureia-Itatíns, Rio Verde, fallen trunk, 28.VI.2017 Pires 406* (H).

*Protohydnumgalzinii* (Bres.) Spirin & R.H. Nilsson. Austria. Niederösterreich: Perchtoldsdorf, Naturpark Föhrenberge, *F.excelsior*, 10.IX.2022 Spirin 15803 (H). France. Alpes-Maritimes: Cannes, Ile de Saint-Marguerite, *Quercusilex*, 12.XI.2022 Gruhn 221112-005, 221112-007 (G.G., H), unidentified angiosperm wood, 12.XI.2022 Gruhn 221112-013 (G.G., H). Aveyron: Combret, Bétirac, *P.nigra*, 21.III.2024 Spirin 17091 (H), *F.excelsior*, 6.XI.2024 Spirin 17818 (H, GB). Drôme: Montélimar, *Platanusorientalis*, 11.XI.1966 Lanquetin (LY JB-5742). Landes: Port-de-Lanne, *A.glutinosa*, 17.III.1985 Gilles 486 (LY JB-10973). Pyrénées-Atlantiques: Pardies, rotten wood, 22.III.1964 Beller (LY JB-4761); Sauveterre-de-Béarn, *P.nigra*, 26.III.1983 Gilles 045 (LY JB-10128), 29.III.1985 Gilles (LY JB-11893). Isère: Veyrins-Thuellin, Les Avenières, *P.nigra*, 29.III.2012 Rivoire (LY BR4339, H). Rhône: Millery, *Hederahelix*, 29.IV.2014 Rivoire (LY BR5393). Iran. Mazanderan: Jangale, fallen branch, 11.VII.1976 Hallenberg 1674 (GB-0185314), fallen decayed branch, 12.VII.1976 Hallenberg 1729 (GB-0185313). Italy. Veneto: Venice, Carpenedo, deciduous wood, 1.V.1992 Losi (GB-0078123). Russia. Krasnodar Reg.: Khosta, Acun, fallen angiosperm log, 17.IX.1996 Hallenberg 13135 (GB-0184376). Spain. Canary Ids.: La Palma, Puntallana, angiosperm wood, 25.VII.2022 Viner 2022/1023* (H). Málaga: Mijas, Los Espartales, on a living tree (Cupressaceae), 20.XI.2012 Miettinen 15900.4* (H). Ukraine. Lugansk Reg.: Stanichno-Luganskii Dist., Severnyi Donets, *Populusalba*, 20.X.2010 Akulov (CWU 4564*).

*Protohydnumlactescens* (Burt) Spirin & V. Malysheva. USA. California: Napa Co., St. Helena, on wood, 29.XII.1960 Wells* (TAAM192048), Sage Creek, on wood, 8.XII.1962 Wells & Prusso (TAAM192047).

*Protohydnumlivescens* (Bres.) Spirin & V. Malysheva. Austria. Niederösterreich: Perchtoldsdorf, Naturpark Föhrenberge, *Pinusnigra*, 10.IX.2022 Spirin 15807, 15810 (H). France. Aveyron: Millau, Le Causse Noir, *P.nigra*, 21.II.2025 Spirin 18076 (H), Le Pas Destrech, *P.nigra*, 7.XI.2024 Spirin 17873 (H). Lozère: Saint-Etienne-du-Valdonnez, Forêt du Sapet, *A.alba*, 28.VI.2022 Spirin 15514, 15530 (H), 29.VI.2022 Spirin 15619, 15622 (H). Rhône: St. Laurent d’Agny, Bois Bouchat, *P.sylvestris*, 8.XII.2020 Rivoire 7684 (LY). Greece. Central Athens: Kaisariani, Asteriou, *Pinushalepensis*, 10.I.2022 Viner 2022/7* (H). Romania. Brașov: Prund-Schei, *A.alba*, 20.V.2017 Miettinen 20660* (H). Slovenia. Gorenjska: Ravne v Bohinju, *A.alba*, 28.VII.2020 Spirin 13886, 13913* (H). Kočevje: Podstenice, Rajhenavski Rog, *A.alba*, 19.VIII.2021 Spirin 14774 (H). Spain. Andalusia: Málaga, Mijas, *P.halepensis*, 16.XI.2012 Miettinen 15891.1* (H), *Pinuspinaster*, 16.XI.2012 Miettinen 15872.2* (H), 24.XI.2012 Miettinen 16000* (H). Ukraine. Zakarpats’ka Reg.: Kuzy, *A.alba*, VIII.1934 Pilát (H ex Fungi Carpatici Lignicoli Exsiccati #152).

*Protohydnumnudum* Spirin & Ryvarden. Kenya. Western Province: Kakamega Forest, decayed wood, 25–27.I.1973 Ryvarden 9457 (O, H).

*Protohydnumpululahuanum* (Pat.) Spirin. Ecuador. Pichincha: Quito, Pululahua, decayed wood, [no collecting date] Lagerheim (FH00783538, 00783539, 00783540).

*Protohydnumsucinum* (Möller) Spirin & Alvarenga. Brazil. [no locality and collecting date], Rick (Lloyd’s herbarium #38807, 33209, as *Auriculariabrasiliensis*) (BPI701264, 701268).

*Protohydnum* sp. French Guiana. Régina: Chute de Patawa, hardwood, 31.VIII.2018 Vlasák 1808/145* (H).

*Protomeruliusamiliavi* Spirin. France. Aveyron: Millau, Le Causse Noir, *Q.pubescens*, 21.II.2025 Spirin 18037 (H).

*Protomeruliusbrachysporus* (Luck-Allen) Spirin & Malysheva. Canada. British Columbia: Vancouver Island, between Cowichan and Port Renfrew, dead wood, 12.VIII.1988 Hallenberg 10651 (GB-0185453). France. Aveyron: Millau, Larzac, Le Rajal del Gorp, *P.sylvestris*, 14.XI.2022 Spirin 16282, 16285, 16288* (H), L’Hospitalet-du-Larzac, *J.communis*, 8.XI.2024 Spirin 17963 (H). Slovenia. Kočevje: Podstenice, Rajhenavski Rog, *A.alba*, 19.VIII.2021 Spirin 14820 (H). Sweden. Gothland: Uppsteig, *P.abies*, 21.X.1984 Hallenberg 8719 (GB-0185439). Uppland: Lundsvedja, strongly rotten wood, 12.VI.1952 Eriksson 6646 (GB-0185443). Västergötland: Medelplana, *P.abies*, 8.X.2008 Larsson 13891 (GB-0087471). Västerbotten: Holmsund, Obbola, *P.abies*, 1.X.1970 Strid 7899 (GB-0185444). Östergötland: Väversunda, Omberg, *Abies* sp., 30.X.1978 Hallingbäck (GB-0185442). USA. California: San Bernardino Nat. Forest, San Jacinto Mts., conifer, 17.III.1984 Ryvarden 21748 (GB-0185452).

*Protomeruliuscommotus* Spirin & V. Malysheva. France. Aveyron: Castelnau-Pégayrols, Le Trou d’Enfer, *Q.pubescens*, 22.III.2024 Spirin 17127* (H). Italy. Liguria: Imperia, Pigna, Buggio, *Carpinusbetulus*, 17.X.2019 Spirin 13835* (H). Norway. Vestfold: Larvik, Jordstøyp i Kvelde, *U.glabra*, 15.IX.2016 Spirin 11110* (O). Switzerland. Ticino: Sementina, Boschetti, *F.excelsior*, 13.X.2019 Spirin 13617* (H). Sweden. Västergötland: Hällekis, Törnsäter Nat. Res., *U.glabra*, 5.X.2024 Spirin 17657 (GB).

*Protomeruliusdeceptorius* Spirin & Viner. Slovenia. Cerknica: Goričice, *S.caprea*, 30.IX.2023 Spirin 16863* (H). Ilirska Bistrica: Snežnik, *F.sylvatica*, 17.VIII.2021 Spirin 14634, 14592*, 14651*, 14652 (H), 28.IX.2023 Spirin 16764*, 16773, 16784, 16788 (H). Kočevje: Borovec pri Kočevski Reki, Krokar Forest Reserve, *F.sylvatica*, 18.VIII.2021 Spirin 14659 (H).

*Protomeruliusdubius* (Bourdot & Galzin) Spirin & Malysheva. Finland. Uusimaa: Helsinki, Veräjämäki, Pirunkallio, *Salix* sp., 22.X.2019 Viner 2019/154* (H). Sweden. Öland: Högsrum, *Q.robur* (rotten log), 18.X.1990 B. Nordén (GB).

*Protomeruliusmadidus* Spirin & K.H. Larss. France. Aveyron: Saint-Sernin-sur-Rance, Le Bouissou, *Q.pubescens*, 6.XI.2024 Spirin 17851* (H). Romania. Braşov: Şinca, *F.sylvatica*, 14.IX.2021 Spirin 14943 (H). Arad: Bârzava, Runcu-Groşi, *F.sylvatica*, 15.IX.2021 Spirin 15021* (H). Russia. Nizhny Novgorod Reg.: Lukoyanov Dist., Razino, *P.tremula*, 27.VII.2019 Spirin 12699 (H), *U.glabra*, 28.VII.2019 Spirin 12724 (H), Sanki, *Ulmuslaevis*, 31.VIII.2019 Spirin 13076 (H), *Q.robur*, 31.VII.2021 Spirin 14455 (H). Sweden. Västergötland: Hällekis, Törnsäter Nat. Res., *C.avellana*, 5.X.2024 Spirin 17651 (GB). Medelplana, Munkägarna, *C.avellana*, 15.X.2023 Spirin 16931 (GB), *U.glabra*, 15.X.2023 Spirin 16926, 16940, 16951*, 16953 (GB). Västmanland: Rytterne, Kalvholmen, *Salix* sp., 28.X.1981 Hallingbäck (GB-0185454). Uppland: Uppsala, Vårdsätra, angiosperm wood, 19.X.1967 Sunhede & Eriksson (GB-0185455). Gästrikland: Gävle, Lövudden, *Betula* sp., 4.VIII.1957 Nannfeldt 15318 (GB-0185456).

*Protomeruliuspertusus* Malysheva & Spirin. Belgium. Antwerp: Wilrijk, Fort 7, deciduous log, 2000 De Meulder (GB-0185448). Norway. Sør-Trøndelag: Trondheim, Almelia, *U.glabra*, 28.VIII.1982 Hjortstam 12902 (GB-0185446). Russia. Adygea: Maykop Dist., Guzeripl, *F.sylvatica*, 17.IX.2003 Kotiranta 22589* (H). Nizhny Novgorod Reg.: Lukoyanov Dist., Razino, *U.glabra*, 28.VII.2019 Spirin 12743* (H).

## Supplementary Material

XML Treatment for Elmericium

XML Treatment for Elmericium alabastrinum

XML Treatment for Hydrophana

XML Treatment for Hydrophana fessula

XML Treatment for Hydrophana trichiesiana

XML Treatment for Mycostilla

XML Treatment for Mycostilla chromatica

XML Treatment for Myxarium

XML Treatment for Myxarium denticulatum

XML Treatment for Myxarium guianense

XML Treatment for Myxarium inconspicuum

XML Treatment for Myxarium legonii

XML Treatment for Myxarium spiniferum

XML Treatment for Protoacia

XML Treatment for Protoacia crispans

XML Treatment for Protoacia reliqua

XML Treatment for Protohydnum

XML Treatment for Protohydnum album

XML Treatment for Protohydnum aureum

XML Treatment for Protohydnum cartilagineum

XML Treatment for Protohydnum elasticum

XML Treatment for Protohydnum elevatum

XML Treatment for Protohydnum erumpens

XML Treatment for Protohydnum galzinii

XML Treatment for Protohydnum glabrum

XML Treatment for Protohydnum lactescens

XML Treatment for Protohydnum livescens

XML Treatment for Protohydnum microperum

XML Treatment for Protohydnum nudum

XML Treatment for Protohydnum ocellatum

XML Treatment for Protohydnum pallidum

XML Treatment for Protohydnum pululahuanum

XML Treatment for Protohydnum sucinum

XML Treatment for Protohydnum

XML Treatment for Protomerulius

XML Treatment for Protomerulius amiliavi

XML Treatment for Protomerulius commotus

XML Treatment for Protomerulius deceptorius

## ﻿Additional information

### Use of AI

No use of AI was reported.

### Author ORCIDs

Renato Lúcio Mendes Alvarenga https://orcid.org/0000-0003-0400-3762

Tine Grebenc https://orcid.org/0000-0003-4035-8587

Gérald Gruhn https://orcid.org/0000-0003-4829-4960

Anton Savchenko https://orcid.org/0000-0001-6377-6349

Django Grootmyers https://orcid.org/0000-0002-1042-4902

Josef Vlasák https://orcid.org/0000-0002-5363-4459

Karl-Henrik Larsson https://orcid.org/0000-0002-1248-3674

R. Henrik Nilsson https://orcid.org/0000-0002-8052-0107

### Data availability

All of the data that support the findings of this study are available in the main text.
